# Methodological differences between studies confound one-size-fits-all approaches to managing surface waterways for food and water safety

**DOI:** 10.1128/aem.01835-23

**Published:** 2024-01-12

**Authors:** Daniel L. Weller, Claire M. Murphy, Tanzy M. T. Love, Michelle D. Danyluk, Laura K. Strawn

**Affiliations:** 1 Department of Biostatistics and Computational Biology, University of Rochester Medical Center, Rochester, New York, USA; 2 Department of Food Science and Technology, Virginia Tech, Blacksburg, Virginia, USA; 3 Department of Food Science and Human Nutrition, Citrus Research and Education Center, University of Florida, Lake Alfred, Florida, USA; The Pennsylvania State University, University Park, Pennsylvania, USA

**Keywords:** produce safety, methods comparison, water quality, *Salmonella*, *Listeria*, shiga toxin* Escherichia coli*

## Abstract

Even though differences in methodology (e.g., sample volume and detection method) have been shown to affect observed microbial water quality, multiple sampling and laboratory protocols continue to be used for water quality monitoring. Research is needed to determine how these differences impact the comparability of findings to generate best management practices and the ability to perform meta-analyses. This study addresses this knowledge gap by compiling and analyzing a data set representing 2,429,990 unique data points on at least one microbial water quality target (e.g., *Salmonella* presence and *Escherichia coli* concentration). Variance partitioning analysis was used to quantify the variance in likelihood of detecting each pathogenic target that was uniquely and jointly attributable to non-methodological versus methodological factors. The strength of the association between microbial water quality and select methodological and non-methodological factors was quantified using conditional forest and regression analysis. Fecal indicator bacteria concentrations were more strongly associated with non-methodological factors than methodological factors based on conditional forest analysis. Variance partitioning analysis could not disentangle non-methodological and methodological signals for pathogenic *Escherichia coli*, *Salmonella*, and *Listeria*. This suggests our current perceptions of foodborne pathogen ecology in water systems are confounded by methodological differences between studies. For example, 31% of total variance in likelihood of *Salmonella* detection was explained by methodological and/or non-methodological factors*,* 18% was jointly attributable to both methodological and non-methodological factors. Only 13% of total variance was uniquely attributable to non-methodological factors for *Salmonella*, highlighting the need for standardization of methods for microbiological water quality testing for comparison across studies.

**IMPORTANCE:**

The microbial ecology of water is already complex, without the added complications of methodological differences between studies. This study highlights the difficulty in comparing water quality data from projects that used different sampling or laboratory methods. These findings have direct implications for end users as there is no clear way to generalize findings in order to characterize broad-scale ecological phenomenon and develop science-based guidance. To best support development of risk assessments and guidance for monitoring and managing waters, data collection and methods need to be standardized across studies. A minimum set of data attributes that all studies should collect and report in a standardized way is needed. Given the diversity of methods used within applied and environmental microbiology, similar studies are needed for other microbiology subfields to ensure that guidance and policy are based on a robust interpretation of the literature.

## INTRODUCTION

Food and waterborne disease outbreaks have been linked to the use of contaminated water for food production or recreation (1–6). There is interest in developing models to predict when, where, and how much water is likely to become contaminated with enteric bacteria and foodborne pathogens. These models may support risk assessments and the development of guidance for monitoring and managing hazards in waters. Model development relies on having sufficiently large quantities of data for model training and testing. A multitude of studies have surveyed waters for foodborne pathogens, pathogen surrogates, and fecal indicator bacteria (7–18). The sampling and laboratory methods used by published studies are diverse and often vary by laboratory and available resources. For example, some studies collected water samples using Moore swabs, which capture microbial water quality flowing through a waterway during a given time frame (7, 16, 19), while other studies collected water using grab samples, which provides a snapshot of water quality at the moment of sample collection (16, 20). For grab samples, volumes vary substantially between studies with ranges between 3.33 mL (20) and 10 L (16, 21). Furthermore, both filtration methods (11, 16, 20, 22–24) and foodborne pathogen detection [culture-based (12, 25, 26) versus molecular-based methods (13, 21)] have varied across studies.

Previous studies have reported that methodological differences affect observed microbial water quality (15, 16, 27–32). The odds of pathogenic *Escherichia coli* and *Salmonella* detection were lower for 10-L grab samples filtered through modified Moore swabs, compared to 24-hour Moore swabs collected from the same waterways at the same time (16). A Mid-Atlantic study reported *Salmonella* detection was 26 and 44 times more likely when 10-L samples were collected compared to 1.0- and 0.1-L samples, respectively (32). Findings from these and other studies demonstrate that results may not be comparable when different sampling and laboratory methods are used. However, these past studies were limited geographically (e.g., focused on one or two regions and sampling a small number of waterways), temporally (e.g., conducted over a single growing season), and/or in sample size (e.g., small number of samples were collected). To address these limitations, the present study assessed how methodological differences impact the comparability of study findings using data from multiple studies to ensure sufficient sample size, geographical diversity, and temporal coverage.

Prior studies have also investigated and compared microbial water quality between water type and region (12, 16, 33–36). A 2020 review of agricultural water in the Southeastern United States noted that the geographical location of a water source played an important role in the prevalence and survival of foodborne pathogens (35). This conclusion is supported by other studies that compared microbial water quality between growing regions nationally (16, 37) and locally (12, 17, 38), and between water types (32, 39–41). Indeed, multiple studies have shown that this variability in water quality limits the efficacy of one-size-fits-all approaches to monitoring and mitigating microbial hazards in aquatic environments. Understanding how microbial water quality differs between water types (e.g., canal, pond, and stream) and regions is needed for the development of water type and regional guidance for managing and mitigating these hazards. Additionally, identification of strong regional and methodological signals by some studies (15, 16, 32) further highlights the challenge of comparing findings between studies conducted in different regions and on different water types when those studies also used different methods. Data are needed to determine how and if (i) such findings can be compared and (ii) methodological signals can be separated from signals of interest (e.g., region and water type).

To address these knowledge gaps, we compiled a large data set representing a diversity of regions, water types, and sampling and laboratory methods. We used these data to (i) quantify the impact of methodological differences on observed microbial water quality; (ii) determine if methodological signals can be disentangled from other signals of interest (e.g., region and water type); and (iii) evaluate how using specific methodological differences (e.g., collecting 1 L versus 10 L of water) impacts observed microbial variability in water. To determine if strategies for managing microbial hazards in surface water should be region, water type, and/or waterway specific, we examined how strongly (i) water type and waterway-specific factors and (ii) regional classification scheme (i.e., how samples were assigned to a given regions, such as by ecoregion or climate region) were associated with microbial water quality after accounting for methodological differences.

## RESULTS

### Data quality and compatibility

**Fig 1:**
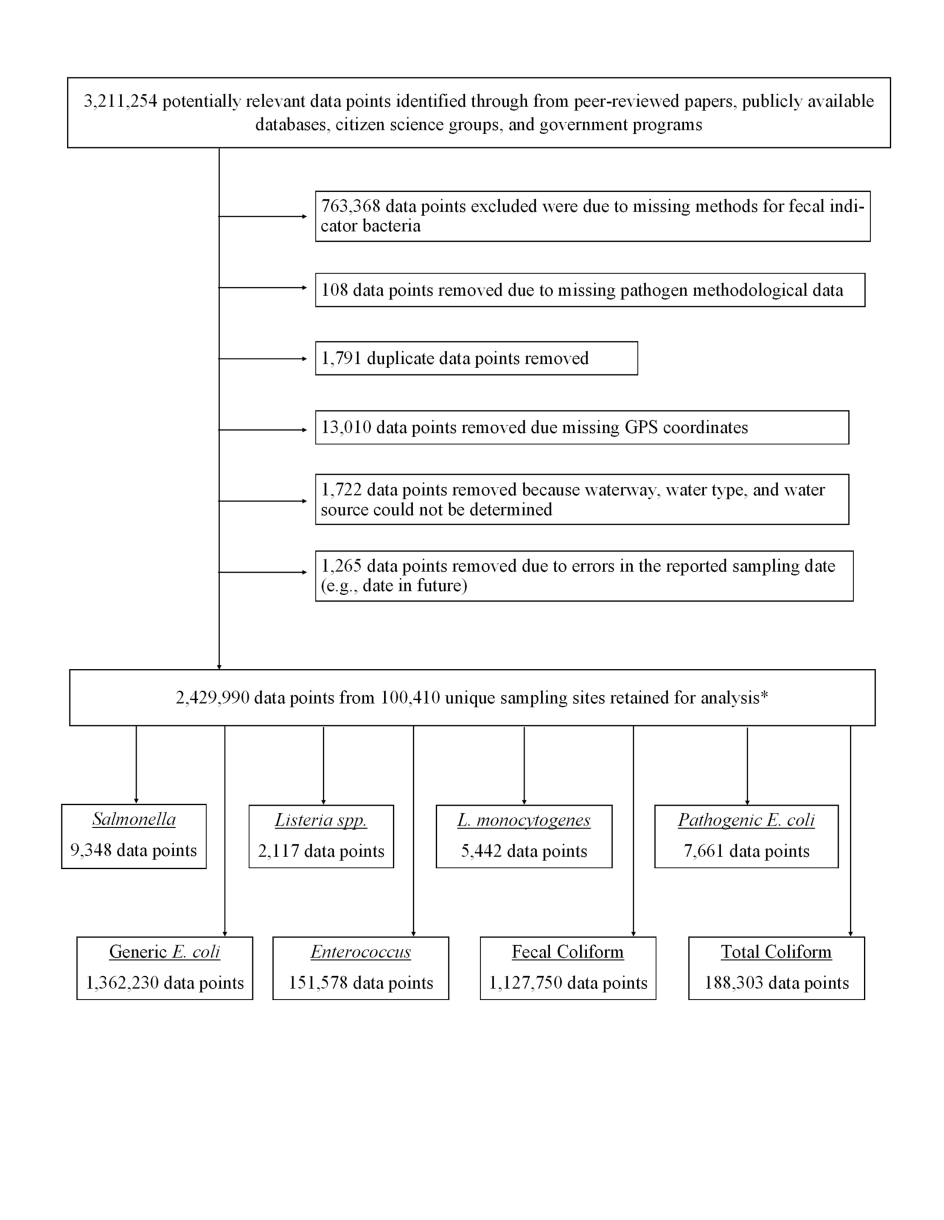
Schematic representation showing data exclusion due to data quality and compatibility issues. *Numerous samples were tested for more than one microbial target. GPS, Global Positioning System.

**Fig 2:**
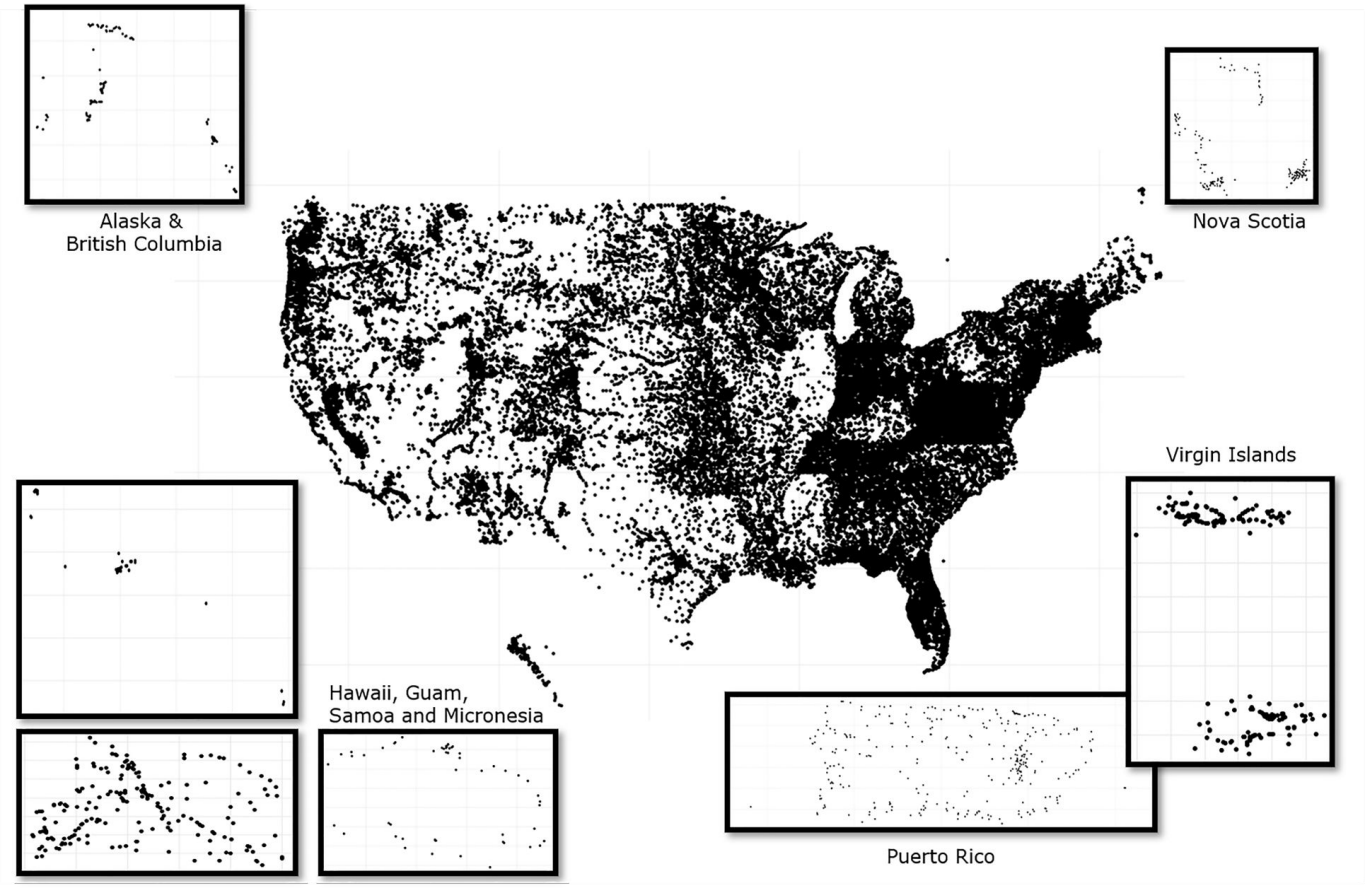
Location of the 100,410 unique sampling site locations represented by the data set compiled here. GPS coordinates were modified slightly to ensure confidentiality. (The maps were created in R using the ggplot2 and sf packages.)

Of the 3,211,254 data points collected from peer-reviewed papers, publicly available databases, citizen science groups, and government organizations, 2,429,990 (77%) were retained in the final data set (Fig. 1), and are also provided in an online database (https://github.com/wellerd2/Weller-et-al-2024-AEM-Datasets/tree/main). The retained datapoints represent 100,410 unique sampling sites and included data points from all US states, as well as multiple US territories, Canadian provinces, and Mexican states (Table 1; Fig. 2; Fig. S1). Additionally, data encompasses numerous major agricultural regions (e.g., the Central Valley, the Columbia Basin, and the Mid-Atlantic). Table 2 was developed to support future field studies on microbial water quality, highlighting recommended practices for data collection, recording, reporting, and future analyses. During data compilation, studies with insufficient methodological data were dropped, such as samples where volume for enumeration was not available. The vast majority of the 781,264 datapoints excluded were because methods for generic *E. coli*, total coliforms, fecal coliforms, or *Enterococcus* did not provide any sufficient information on sampling and/or enumeration methods (Fig. 1). One hundred and eight datapoints of foodborne pathogen data were excluded because methodological data were not available. Since complete GPS coordinates were needed to extract key non-methodological data (e.g., water type) assign unique site and waterway IDs, and apply the regional classification schemes, 13,010 datapoints were excluded where complete GPS coordinates were not reported or had missing information. Other datapoints that were excluded include 1,791 duplicates and 1,722 datapoints where waterway, water type, and water source could not be determined. Errors in sampling date resulted in 1,265 datapoints being excluded; most of these were excluded because the reported sample collection was listed as a year in the future or unrealistically far in the past. The vast majority of excluded datapoints were collected by local, state, or government agencies and were downloaded from government portals. Almost all data provided directly by research or citizen science groups or obtained from peer-reviewed papers were retained (Fig. 1).

**TABLE 1:** Summary of the data sets compiled as part of the study presented here^*h*^

Data source^*a*^	Organization type^*b*^	Years^*c*^	State^*d*^	No. of samples tested for^*e*^							Data sets^*f*^	Citation^*g*^
				Sites	Total samples	Fecal indicator bacteria						
						Coliforms		*E. coli*	*Enterococcus*	Pathogens		
						Fecal	Total					
ACAP, St. John	N/C	1995, 2019	NB	96	1,473	1,473	0	0	0	0	ACAP	(42)
Adhikari Lab, LA St U	U	2017–2018	LA	1	29	0	0	29	0	0	ADH	(43)
Bhullar Lab, KS St U	U	2017–2020	IA, KS, MO	511	511	0	0	511	0	0	BHU, KSM	(44, 45)
Black Warrior Riverkeeper	N/C	2019–2020	AL	9	83	0	1	83	0	0	BWR	(46)
CA Env Data Exchange Network	G	2009, 2019	CA	25	456	230	46	44	220	0	CEDEN	(47)
Canizalez-Roman Lab, U Autonoma de Sinaloa	U	2015	SI	173	405	118	393	390	0	405	ANF	(20)
Cary Inst for Ecosystem Studies	U	2001, 2017	MD	12	1,702	0	0	1,702	0	0	BES	(48)
Characklis Lab, U of NC at Chapel Hill	U	2004, 2008	NC	11	256	209	0	250	152	85	UNC, UNCS	(49–51)
Chesapeake Bay Foundation	N/C	2015–2016	MD, PA, and VA	64	591	50	0	193	398	0	CBF	(52)
Chesapeake Monitoring Cooperative	N/C	2011–2020	DC, DE, MD, and VA	415	8,383	91	0	7,889	1,463	0	CMC	(53)
City of Austin, TX	G	1997, 2021	TX	311	6,059	0	0	6,059	0	0	AUST	(54)
City of Chicago, IL	G	2006–2016	IL	25	17,598	0	0	2,634	14,964	0	BLD	(55)
Colwell Lab, U of MD	U	2019	DC	4	8	0	0	8	8	8	METG	(56)
Community Sci Inst	N/C	2002–2021	NY	273	6,511	0	4,541	6,454	0	0	CSI	(41)
Danyluk Lab, U of FL	U	2010–2016	FL	39	910	0	910	910	540	910	NUF, RUF, ZUF	(11–13)
Dept of the Env, Prince George’s Co, MD	G	2008–2020	MD	2	402	34	0	368	0	0	BEAR	-
Food Safety Lab, Cornell U	U	2001, 2018	AZ, CA, and NY	587	1,745	0	800	800	0	1,730	FSL, PAWQ	(9, 15, 16, 33, 34, 36, 57–60)
Four Rivers Watershed Watch	N/C	2001–2018	KY and TN	279	2,769	543	0	2,226	0	0	FRSS	(61)
GA Env Monitoring and Assessment System	G	1999–2021	GA and SC	737	21,200	19,599	0	6,472	788	0	GOMAS	(62)
Green Lab, SUNY College of Env Sci and Forestry	U	2015, 2020	NY and UT	99	693	0	659	654	29	0	ESF, SLC	(63)
Hansen Lab, Dalhousie U	U	2008–2009	NS	12	333	0	333	333	0	333	LTH	(23)
Harwood Lab, U of South FL	U	2009–2012	FL	12	84	84	0	0	84	0	ZRS	(64)
Hornor Lab, Anne Arundel Co Community College	U	2001, 2019	MD	8	152	0	0	0	152	0	OPS	-
IA Dept of Natural Resources	G	1991–2020	IA	60	14,613	3,791	0	13,919	3,096	0	IDNR	(65)
Levy Lab, Emory U	U	2013	GA	16	107	0	0	105	0	107	CSH	(22)
LA Dept of Env Quality	G	1978, 2020	LA	9	1,205	1,205	0	0	0	0	LADEQ	(66)
Lower Colorado River Authority	G	1982–2019	TX	291	16,645	8,815	0	9,669	305	0	TXS	(67)
McLellan Lab, U of WI	U	2011–2013	MI, OH, and WI	30	539	319	0	536	537	0	GRTLK, MILK	(68, 69)
Milwaukee Riverkeeper	N/C	2014, 2019	WI	195	5,131	4,562	0	3,266	0	0	MRK	(70)
Mountain True	N/C	2018–2020	NC and TN	75	1,100	0	0	1,100	0	0	BRW, FBR	(71)
Nashwaak Watershed Watch	N/C	1996, 2019	NB	27	334	0	0	334	0	0	NASH	(72)
National Water Quality Portal	G	1919, 2022	^*g*^	79,121	2,141,249	961,883	155,571	1,257,650	99,214	1,979	^*f*^	(73)
OK Dept of Ag	G	2001–2014	MO and OK	275	4,599	3,459	0	3,832	3,867	0	OKW	(74)
OK Water Survey, U Of OK	U	2018	OK	24	306	0	0	296	273	0	OWS	(75)
Onondaga Env Inst	N/C	2008, 2017	NY	180	2,658	2,658	96	281	288	0	OEI	(17)
Pearl Riverkeeper	N/C	2018–2020	LA and MS	31	428	428	419	426	0	0	PRWS	(76)
Pickering Lab, Tufts U	U	2017	MA	2	4	0	0	0	0	4	PICK	(77)
Public Health Lab, Humboldt Co, CA/U of South FL	G, U	2020	CA	13	201	0	201	201	201	0	STRAWB	(78)
Richardson Lab, Cornell U	U	2017–2018	NJ and NY	36	179	0	0	163	174	65	RICH	(21, 79)
Rideout Lab, VA Tech	U	2013–2015	VA	4	434	0	191	191	0	431	GUF	(80)
Rock Lab, U of AZ	U	2012	AZ and CA	204	446	0	253	446	0	0	CPS	(81)
Saint Croix International Waterway Commission	N/C	1998, 2019	NB	96	245	0	0	245	0	0	SCIWC	(82)
Shariat Lab, U of GA	U	2018–2019	PA	28	112	0	111	111	0	112	SRB	(25)
Shrestha Lab, U of IL at Chicago	U	2016	IL	7	195	0	0	170	195	0	SHR	(83)
Smith Mountain Lake MP, Ferrum College	U	1995–2020	VA	26	4,068	0	4,066	2,681	0	0	FERR	(84, 85)
SC Adopt-a-Stream, Clemson U	U, N/C	2016–2020	SC	201	1,435	0	0	1,435	0	0	SCAAS	(86)
Spa Creek Conservancy	N/C	2016–2020	MD	16	672	0	0	0	672	0	SCC	(87)
Strawn Lab, VA Tech	U	2015, 2021	VA	63	1,120	0	1,000	1,000	0	520	DUCK, LW, TUF	(12, 14, 38, 88)
Surface Water Ambient MP, CA Water Boards	G	2018–2021	CA	26	1,992	0	0	1,992	0	0	SWA, SWAMP	(89)
Thornton Creek Alliance	N/C	2017–2020	WA	24	2,005	0	0	2,005	0	0	THC	(90)
US Dept of Ag	G	2011–2016	CA and IA	22	3,164	22	3,164	58	22	3,164	CEG, CLY, NASA	(7, 19, 91)
US Env Prot Agency	G	2012–2017	^*i*^	5,396	8,200	0	1,107	2,189	7,053	126	BRAD, CLCR, NARS, SFBR	(31, 92–96)
US Geological Survey	G	2001–2019	^*j*^	315	8,387	226	1,273	7,191	764	809	^*k*^	(24, 97–123)
Waccamaw Watershed Academy, Coastal Carolina U	U	2008–2020	NC and SC	28	7,364	1,322	6,240	6,242	0	0	CCU	(124)
Walters Lab, Stanford U	U	2008–2009	CA	14	241	0	0	241	227	241	SPW	(26)
Wang Lab, U of BC	U	2015–2016	BC	7	446	411	0	397	0	446	UBC	(125)
WA Dept of Ag	G	2014–2021	WA	823	41,184	38,985	0	2,201	0	0	WAWA	(126)
Watershed Watch, U of RI	U	1991, 2020	CT, MA, NY, and RI	560	19,508	10,335	0	2,866	15,892	0	URI	(127, 128)
WV Dept of Env Prot	G	1970–2020	WV	8,458	67,038	66,898	6,928	699	0	0	WVD	(129)
Western Center for Food Safety, U of CA at Davis	U	2012–2013	GA	2	83	0	0	83	0	0	EMA	(130)

^*a*^ ACAP, Atlantic Coastal Action Program; Ag, Agriculture; Co, County; Dept, Department; Env, Environment/Environmental; Inst, Institute; MP, Monitoring Program; Prot, Protection; Sci, Science; St, State; U, University.

^*b*^ Organization type: G, governmental organization; N/C, non-profit or citizen science organization; U, university.

^*c*^ The first and last years that data from the given organization were available. Some organizations collected data every year during this period (indicated by the use of “–”), while other organizations did not continuously collect data during this period (indicated by the use of “,”).

^*d*^ US Post Office abbreviations are used for Canadian provinces, Mexican states, and US states and territories.

^*e*^ The number of samples with data on fecal indicator bacteria concentrations and the presence/absence of any of the foodborne pathogens (e.g., *Listeria monocytogenes* and *Salmonella*) or indicator organisms (e.g., *Listeria* spp.) for foodborne pathogens.

^*f*^ Some groups or databases provided data from multiple data sets. These codes correspond to each data set provided by each group or database except for the National Water Quality Portal (NWQP); these codes are used to link information in Supplemental Table 2. Because of the number of data sets available in and downloaded from the NWQP (*N*=534), all NWQP data sets could not be listed here.

^*g*^ Data and attributes were downloaded from publicly available portals and peer-reviewed papers, or obtained through personal communication with project leads or points of contacts in the relevant organization, lab, or agency. Groups with no web presence or corresponding report/publication that were contacted by email are indicated by “–.”

^*h*^ Data were available for all 50 US states as well as American Samoa, British Columbia, the District of Columbia, Guam, Puerto Rico, Saskatchewan, and the US Virgin Islands.

^*i*^ Data were available for the 48 continental United States but not for AK or HI.

^*j*^ Data were available for DE, GA, IA, IL, IN, KS, KY, MI, MO, NC, NJ, OH, OR, PA, RI, SC, SD, TN, TX, VA, and WI.

^*k*^ The datasets available from the US Geological Survey were assigned the following codes: AMRF; BCB; CHAT; CNP; DEGR; DSF; ERIE; FUR; INDPC; LAMI; LCM; LSC; MATH; MIR; MMF; RACI; SALAD; SCIT; SHV; ULRB; UPDU; USGS; WHCH; WHSKR.

**TABLE 2:** Recommended practices for the collection, recording, and reporting of data and attributes for research collecting water samples aimed at understanding microbial water quality

Category	Best practices for collection, recording, and reporting	Resources and references
Tidy data^*a*^		(131, 132)
Data structure	Each column represents a unique variable with a unique column header that avoids spaces, capital letters, or special characters (e.g., water_type or watertype).	
	Each row represents a unique observation with a unique row ID.	
	Each cell is a single measurement without units within the cell (include units in data dictionary and, if needed, create a new column for units).	
	Avoid visual formatting (e.g., colored cells and borders).	
Value formatting	For categorical variables, use drop-down menus when possible so you are only selecting from pre-standardized categories. If drop-down menus are not possible, ensure consistency in value formatting and the case of the text (e.g., avoid using “Pond”, “pond”, and “P” to all refer to pond samples). Ensure consistency in spelling and using of white space/special characters (e.g., avoid “pond”).	
	For numeric, set upper and lower bounds so you can catch entry or measurement errors.	
Missing values	Encode missing values as NA or as a blank. This will ensure it is read as missing data by analysis programs. Do not encode missing value as a number (e.g., 0 or 999), character (e.g., –), or word (e.g., missing).	
Data dictionary^*b*^	Include the column header exactly as seen in the data set.	(133–135)
	Define each variable, including all possible entries/factor levels and their meaning, range of possible numeric values, or accepted values for the variable (e.g., ≥0 and ≤100), and units (when applicable). If a numeric variable has upper and lower limits of detection, report these and how they should be dealt with for analysis.	
	If any imputation or data transformations were or should be employed prior to use, describe these.	
	If the variable can be used alone or in conjunction with other columns to calculate new columns, explain this as well.	
Recommended minimum collected and recorded attributes		
Methodology		(136–140)
Sample type	Examples include grab sample and Moore swab.	
Sample volume	Water volume tested for the given target, not the volume of the sample; these may be the same but often the sample is divided into aliquots used to test for different targets. Ensure all data are entered in the same units [e.g., most probable number (MPN)/100 mL, CFU/mL)	
Sample site	Ideally, this would be at least three columns: the sampling site latitude, the sampling site longitude, and a descriptive column (e.g., near uptake pump or 1 m from into pond from pump). Additional columns that are recommended are sample depth and distance from shore.	
Unique sampler ID	The person who collected the sample; if multiple individuals have the same initials, do not use initials for this column. Use an unambiguous method for this ID.	
Detection or quantification method	If only one method is used throughout the study, clearly state the method used for target detection or enumeration in the data dictionary. The description should provide sufficient detail [e.g., was it a culture-based or molecular method? what was the detection limit(s)? if and how the sample was filtered (e.g., membrane, none, or modified Moore swab), if and how the target was enumerated (e.g., most probable number-based approach or membrane filter-based approach), volume used for reporting results (e.g., count per 100 mL or count per 10 mL)].	
	If there is a standard name for the method [e.g., Environmental Protection Agency (EPA) Method 1603], include that. Do not use organization or lab-specific names in this column, as this information will not mean anything to folks outside the organization or lab.	
	Be clear what the target is (e.g., is it the microbe, a specific gene, or multiple genes?).	
	If the method has previously been validated/published in the peer-reviewed research literature, include this reference. Even if it was published, include key performance information for the method (e.g., sensitivity and specificity).	
	If positive and negative controls were used, include what those controls were.	
Spatial		
GPS coordinates	Use a consistent coordinate reference system (i.e., DATUM) and note this in the data dictionary.	(133, 140–143)
	Use a standard format; formats that include spaces, multiple symbols, or multiple “.” can result in coordinates reading incorrectly. Confirm the appropriate presence/absence of “–” if that is part of the format you use. DO NOT drop the “–” just because all of your sites are in the same hemisphere.	
	Include longitude and latitude as separate columns	
Hydrologic units (HUC)	Six or nine-digit code	
Type of waterway	Examples include pond, stream, or canal.	
Location	This could include separate columns for county, state, county, and/or city.	
Unique site ID	Make it unique to each site and unambiguous. Use a standard way of naming that is relevant to your study design.	
Waterway name	Common name(s) used to refer to the waterway. If multiple names, separate by “;” or “,”.	
Temporal		
Date	MM/DD/YYYY	(138, 140, 144)
Time of day	Use military time (24 hours) to reduce the risk of incorrectly specifying a.m./p.m.	
Physiochemical		
Turbidity	For categorical variables, use a drop-down menu to avoid spelling or entry errors. For numeric values, use a consistent number of significant digits reflects the accuracy of measuring the device and sets minimum and maximum thresholds to catch entry/measurement errors. Use consistent units (e.g., metric or imperial) and include units in the data dictionary.	(138, 145–147)
Water temperature		
pH		
Total suspended solids		
Dissolved oxygen		
Dissolved organic matter		
Conductivity		
Salinity		
Meteorological		
Air temperature	For categorical variables, use a drop-down menu to avoid spelling or entry errors. For numeric values, using a consistent number of significant digits reflects the accuracy of measuring the device and sets minimum and maximum thresholds to catch entry/measurement errors. Use consistent units (e.g., metric or imperial) and include units in the data dictionary.	(138, 147, 148)
Precipitation volume		
Relative humidity		
UV intensity		
Wind speed		

^*a*^ Tidy data provide a standard way to organize data values within a data set that has been cleaned in a way that is ready for analysis.

^*b*^ A data dictionary is a centralized repository of attributes that provides a comprehensive description of the data used. Its main purpose is to provide additional context and information about each data point so that analysts can understand the data better.

Data compilation was complicated by challenges associated with translating data sheets into English. Similarly, the failure of multiple citizen science and government databases to provide method information or list methods online complicated data compilation. These challenges were substantially reduced by the willingness of the researchers to answer questions and share comprehensive data dictionaries as well as reports/certifications found online. Inconsistency in reporting methods information was also discovered. Many citizen science and government data sets used a standard or government method. While peer-reviewed papers often described their methods, these descriptions were often brief and seldom referred to the established methods by name. We also found that many enumeration protocols had multiple names (e.g., Standard Method 9223, Colilert, and Colisure refer to the same protocol). The use of internal laboratory nomenclature or abbreviations required extensive effort to link back to the published protocol. For instance, data available through the US National Water Quality Portal used >15 different United States Geological Survey (USGS)-specific terms to refer to the same protocol. Additional common errors associated with data included latitude as longitude and vice versa, not including the negative sign in the longitude, and reporting unrealistic values for attributes (e.g., pH >14).

Inconsistency in reporting methods and waterway information between and within organizations meant that waterway and water source characteristics had to be verified and corrected. For example, a single data set used >100 different names to refer to a single waterway. Waterway names are not unique to individual waterways, and multiple distinct streams, ponds, or rivers, even in a small geographical area, were identified with the same name. Waterway name categories that were commonly shared between and within geographical locations included color-based (e.g., silver lake), animal-based (e.g., deer creek), person-based (e.g., miller creek), and place-based (e.g., schoolhouse creek) names. For example, it is important to ensure that silver lake in State A will not be coded as the same waterway as silver lake in State B (color-based name). Here, any issues with sampling site ID resulted in its replacement with a geo-ID to avoid confusion.

### Non-methodological and methodological signals could not be disentangled for foodborne pathogens and indicator organisms

#### Salmonella

*Salmonella* data (*N* = 9,348) were obtained from 35 studies representing 15 US states and the District of Colombia, two Canadian provinces, and one Mexican state (Table 1; Table S2). While samples were collected between 1973 and 2019, 75% (*N* = 7,043) were collected since 2012. Grab samples and Moore swabs represented 67% (*N* = 6,245) and 33% (*N* = 3,103) of datapoints, respectively. Of the grab samples, 2,282 (37%) were filtered using membrane filters; 748 (12%) were filtered through modified Moore swabs; 202 (3%) underwent tangential flow filtration; 1,944 (31%) were not filtered; and 1,069 (17%) samples did not describe a filtration method. Culture-based methods were used to detect *Salmonella* in 7,225 samples (77%), while molecular methods were used for 2,123 samples (23%). Seventy percent of studies (*N* = 5,075) that used a culture-based detection method used *invA* to confirm isolates as *Salmonella*.

**Fig 3:**
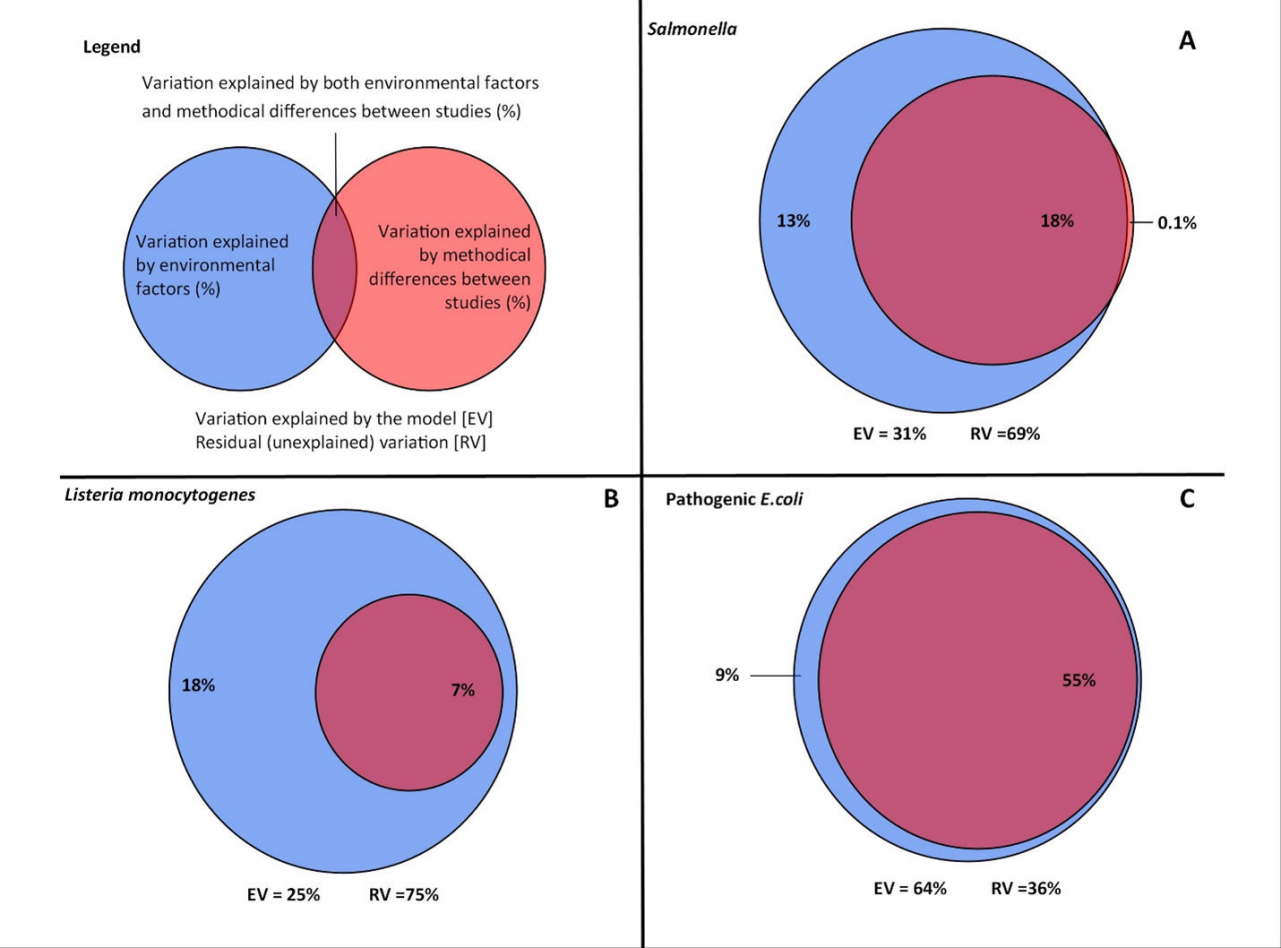
Variance in the likelihood of detecting (**A**) *Salmonella,* (**B**) *Listeria monocytogenes*, (**C**) and pathogenic *E. coli* that is jointly versus uniquely attributable to non-methodological (e.g., sampling site, season, water type, waterway, and year) and methodological (e.g., culture versus molecular-based detection, sample type, and volume) matrices.

Variance partitioning analysis demonstrated that 18% of variance in *Salmonella* detection was jointly attributable to methodological and non-methodological factors, while <1% and 13% of variance was uniquely attributable to methodological and non-methodological factors, respectively (Fig. 3A; Table S3). When variance attributable to waterway/sampling site, year/season, region, and methods was considered, the only source of unique variance greater than 0.01% was waterway/sampling site (4%). When the analysis was done to include waterway/sampling site with year/season, water type, and methodological differences, 10% of variance was jointly attributable to all four sources, while only 6% and 1% were uniquely attributable to waterway/sampling site and methodological differences, respectively; no variance was uniquely attributable to year/season and water type (Table S3).

**Fig 4:**
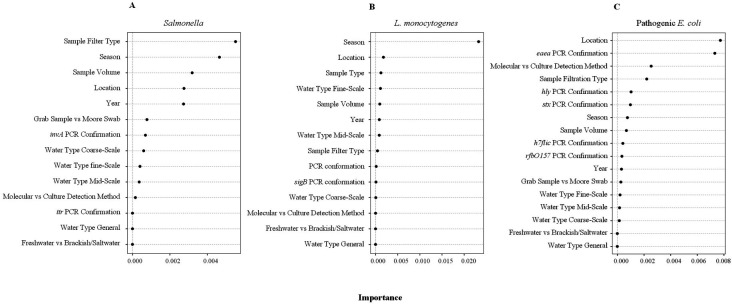
Results of conditional forest analysis that identified methodological and spatiotemporal factors associated with detection of (**A**) *Salmonella*, (**B**) *L. monocytogenes* and (**C**) pathogenic *E. coli* in water. The outcome of these forests was the residuals of a regression analysis that modeled likelihood of target pathogen detection as a function of two nested random effects (site and waterway). The *y*-axis shows the features ranked from highest to lowest variable importance. Variable importance is a unitless relative measure; thus, the importance of one variable should only be compared to another variable in the same plot, not between.

**Fig 5:**
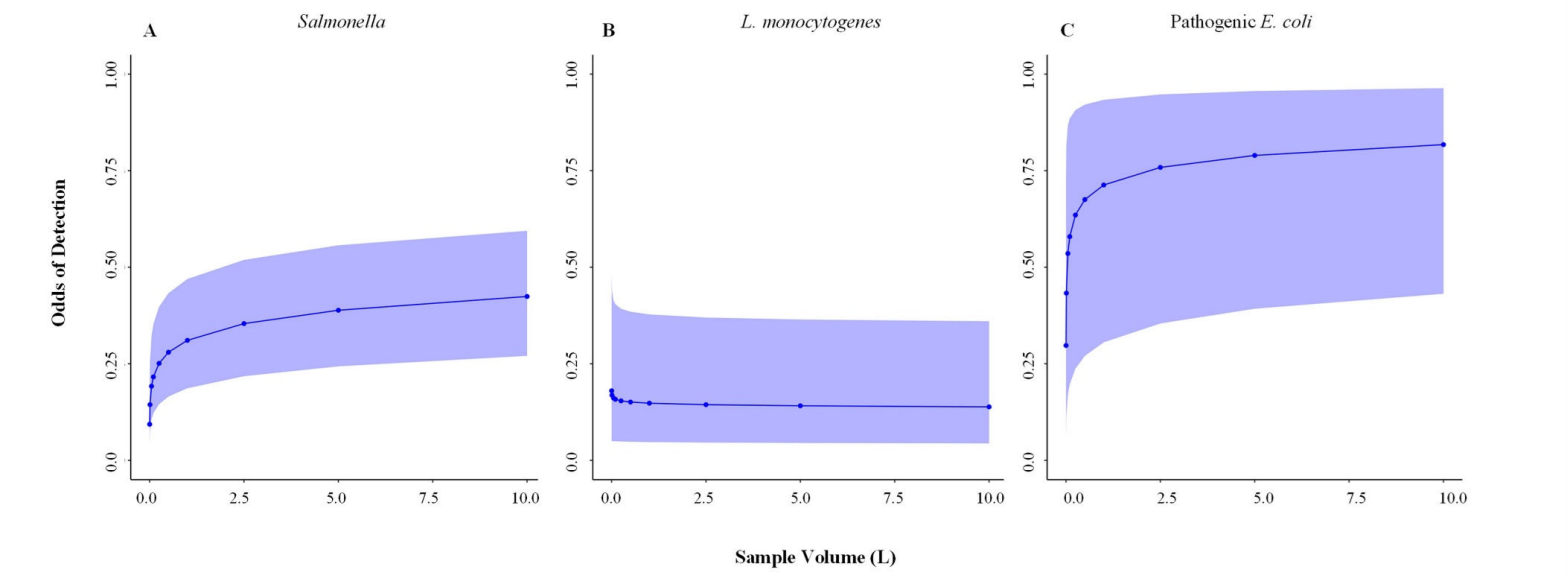
Impact of sample volume on probability of detection of (**A**) *Salmonella*, (**B**) *L. monocytogenes*, and (**C**) pathogenic *E. coli* according to generalized linear mixed models implemented with fixed effects of sample volume and season and random effects of site nested in waterway nested in state. No grab samples were tested for pathogen data in volumes greater than 10 L, and Moore swab volume was set to 10 L.

After accounting for waterway and site-specific signals, the top-ranked factors associated with the likelihood of *Salmonella* detection by conditional forest analysis were sample filtration method, season, and sample volume (Fig. 4A; Table S4). Based on generalized linear models and post hoc testing, the odds of *Salmonella* detection differed significantly between all filtration methods (Table S5). When grab samples were filtered through membrane filters as opposed to modified Moore swabs, there was a fivefold [odds ratio (OR) = 4.70, standard errer (SE) = 1.26; *P < 0.001*] higher odds of *Salmonella* detection (Table S5). Increasing sample volume also significantly increased the odds of detecting *Salmonella.* As volume increased from 5 mL (OR = 0.13, 95% CI = 0.07–0.23) to 10 L (OR = 0.42, 95% CI = 0.27–0.59), *Salmonella* detection increased dramatically (Fig. 5A). Similar differences were observed when different sample types and detection methods were used (Table S5). After accounting for methodological, waterway, and site-specific signals, the top-ranked factors associated with the likelihood of *Salmonella* detection were ecoregion, state, and water type (Table S6).

#### *Listeria* spp. and *L. monocytogenes*

*Listeria* spp. data (*N* = 2,117) were obtained from five studies representing four US states and one Canadian province (Table 1; Table S2). *L. monocytogenes* data (*N* = 5,442) were obtained from eight studies representing four US states and two Canadian provinces (Table 1; Table S2). All *Listeria* spp. and *L. monocytogenes* data were collected between 2001 and 2018. Ninety-six percent of *Listeria* spp. data (*N* = 2,031) and 44% of *L. monocytogenes* data (*N* = 2,398) were grab samples (Table S2). Seventy-seven percent (*N* = 1,573) and 23% (*N* = 458) of grab samples tested for *Listeria* spp. were filtered through modified Moore swabs and membrane filters, respectively, while 19% (*N* = 457) and 79% (*N* = 1,893) of grab samples tested for *L. monocytogenes* were filtered through modified Moore swabs and membrane filters, respectively (Table S2). Two percent (*N* = 48) of grab samples tested for *L. monocytogenes* were not filtered. Culture-based methods were used by all studies to detect *Listeria* spp. and by seven studies (*N* = 5,406) to detect *L. monocytogenes*. Six studies (*N* = 4,060) confirmed isolates as *L. monocytogenes* through PCR and sequencing of *sigB*; one study (*N* = 36) used PCR and sequencing of *hly*; and one study (*N* = 446) used biochemical assays (Table S2).

Only 1% of variance in the likelihood of detecting *Listeria* spp. was jointly attributable to methodological and non-methodological factors, while 2% and 27% of variance were uniquely attributable to methodological and non-methodological factors, respectively (Table S3). Conversely, 8% of variance in the likelihood of detecting *L. monocytogenes* was jointly attributable to methodological and non-methodological factors, while 0% and 18% of variance were uniquely attributable to methodological and non-methodological factors, respectively (Fig. 3B; Table S3).

After accounting for waterway and site-specific signals, the top-ranked factors associated with likelihood of detecting both *Listeria* spp. and *L. monocytogenes* were season, state, and sample type (Fig. 4B; Table S4). Based on generalized linear models and post hoc testing, the odds of isolating *L. monocytogenes* differed significantly between samples that were filtered through any type of filter compared to samples that were not filtered (Table S5). Sample volume was negatively associated with odds of *L. monocytogenes* isolation (OR = 0.92, SE = 0.11, *P* = 0.047) (Fig. 5B; Table S5). After accounting for methodological, waterway, and site-specific signals, the top-ranked factors associated with the likelihood of *Listeria* spp. detection were water type, state, and census region. The top-ranked factors associated with the likelihood of *L. monocytogenes* detection were water type, state, and EPA region (Table S6).

#### Pathogenic *E. coli*

Pathogenic *E. coli* data (*N* = 7,661) were obtained from 19 studies representing 20 US states, 2 Canadian provinces, and 1 Mexican state (Table 1; Table S2). All samples were collected between 2001 and 2019. Approximately half of samples tested for pathogenic *E. coli* were sampled using Moore swabs (*N* = 3,230) and grab samples (*N* = 4,431). Of the grab samples tested for pathogenic *E. coli*, 64% (*N* = 2,834) and 14% (*N* = 642) were filtered through membrane filters and modified Moore swabs, respectively, while 22% (*N* = 955) were not filtered. Culture-based methods were used to detect pathogenic *E. coli* in 58% (*N* = 4,407) of the samples, while molecular methods were used for 42% (*N* = 3,254).

Based on variance partitioning analysis*,* 55% of variance in likelihood of detecting pathogenic *E. coli* was jointly attributable to methodological and non-methodological factors, with 0% and 9% of variance uniquely attributable to methodological and non-methodological factors, respectively (Fig. 3C; Table S3). When the variance in likelihood of detecting pathogenic *E. coli* attributable to waterway/sampling site, year/season, region, and methodological differences was considered, no variance was jointly attributable to all four sources, but 58% was jointly attributable to methods and at least one other source (Table S3). For example, 48% of variance in the likelihood of detecting pathogenic *E. coli* was jointly attributable to method, region, and waterway/sampling site. After accounting for waterway and site-specific signals, the top-ranked factors associated with the likelihood of pathogenic *E. coli* detection were state, the use of *eaeA* for detection/confirmation, and use of culture-based versus molecular detection methods (Fig. 4C; Table S4). Based on generalized linear mixed models and post hoc testing, the odds of pathogenic *E. coli* detection differed significantly (*P* < 0.05) by the genes used for detection/confirmation, if culture or molecular-based methods were used, sample filtration method, and sample volume (Table S5). After accounting for methodological, waterway, and site-specific signals, the top-ranked factors associated with likelihood of pathogenic *E. coli* detection were state, water type, and agricultural region (Table S6).

From the pathogenic *E. coli* samples, 1,978 samples were tested for *eaeA* allowing for identification of enteropathogenic *E. coli* (EPEC). The majority of samples tested for *eaeA* (95.5%; *N* = 1,890) were grab samples (Table S2). Based on variance partitioning analysis, 47% of variance in the likelihood of detecting EPEC was jointly attributable to methodological and non-methodological factors, while <1% and 15% of variance was uniquely attributable to methodological and non-methodological factors, respectively (Table S3). Season, state, and water type (general) were the top-ranked factors associated with EPEC detection (Table S4). In the forest that included regional factors, the highest-ranked factors associated with EPEC were water type, aquatic habitat type, and United States Department of Agriculture (USDA) region (Table S6).

Of the 7,661 samples tested for pathogenic *E. coli*, 6,589 were tested for *stx1* or *stx2* for identification of samples that were presumptively positive for Shiga toxin-producing *E. coli* (STEC; Table 1; Table S2). Approximately half of samples tested for STEC were sampled using Moore swabs (*N* = 3,230) and half were sampled using grab samples (*N* = 3,359). According to variable partitioning analysis, 16% of variance in the likelihood of detecting STEC was jointly attributable to methodological and non-methodological factors, while <1% and 21% of variance was uniquely attributable to methodological and non-methodological factors, respectively (Table S3). After accounting for waterway and site-specific signals, the top-ranked factors associated with likelihood of STEC detection were state, use of culture- versus molecular-based detection methods, and season (Table S4). After accounting for methodological, waterway, and site-specific signals, the top-ranked factors associated with the likelihood of STEC detection were water type, state, and EPA region (Table S6).

Of the 7,661 samples tested for pathogenic *E. coli*, 1,370 grab samples were tested for multiple genes allowing for presumptive identification of *E. coli* O157 positive samples (Table 1; Table S2). After accounting for waterway and site-specific signals, the top-ranked factors associated with likelihood of *E. coli* O157 detection were year, season, and water type (Table S4). After accounting for methodological, waterway, and site-specific signals, the top-ranked factors associated with the likelihood of *E. coli* O157 detection were aquatic habitat, USDA region, and agricultural region.

### Non-methodological factors, as opposed to methodological factors, were more strongly associated with fecal indicator bacteria concentration

Generic *E. coli* (*N* = 1,362,230), *Enterococcus* (*N* = 151,578), fecal coliform (*N* = 1,127,750), and total coliform (*N* = 188,303) data were obtained from 57 US states and territories, Canadian provinces, and Mexican states (Table 1). While data on all four fecal indicator bacteria were collected from a diversity of water types, the proportion of data represented by each water type varied by indicator. For instance, oceans, tidal rivers, and estuaries represented 1% (*N* = 18,157) of *E. coli* data, but 8% (*N* = 12,108), 13% (*N* = 24,288), and 40% (*N* = 456,783) of *Enterococcus*, total coliform, and fecal coliform data, respectively. Conversely, ponds and lakes represented 5% (*N* = 53,818), 13% (*N* = 25,006), 21% (*N* = 38,309), and 24% (328,887) of fecal coliform, total coliform, *Enterococcus*, and *E. coli* data, respectively. Canals represented <5% of data for all four fecal indicators.

While *E. coli* concentrations were enumerated using MPN-based methods for 66% of samples (*N* = 918,919), 30% (*N* = 422,429), 3% (*N* = 48,585), and <1% (*N* = 1,464) were enumerated using membrane filtration, direct plating, or quantitative PCR-based approaches, respectively. IDEXX Quanti-Tray (65%; *N* = 904,814), EPA Method 1103 (10%; *N* = 133,633), and EPA Method 1603 (8%; *N* = 106,500) were most frequently used for *E. coli* enumeration. Total coliform enumeration was most frequently performed using MPN-based methods (69%; *N* = 128,877), membrane filtration (23%; *N* = 43,120), and direct plating (7%; *N* = 13,562). Fecal coliform enumeration was most frequently performed using membrane filtration (58%; *N* = 650,123) and MPN-based methods (43%; *N* = 484,834). Standard Method 9222D was most frequently used for fecal coliform enumeration (54%; *N* = 610,324) followed by AOAC 978 (22%; *N* = 249,272) and APHA 3.2B (9%; *N* = 106,737). Approximately 40% of samples were tested for *Enterococcus* using membrane filtration (*N* = 66,455) and MPN-based methods (*N* = 64,393), while 14% (*N* = 20,730) used a molecular approach. IDEXX Enterolert (50%; *N* = 60,313), EPA Method 1600 (24%; *N* = 36,097), and Standard Method 9230C (20%; *N* = 29,581) were most frequently used for *Enterococcus* enumeration.

After accounting for waterway and site-specific signals, season was the top-ranked factor associated with *E. coli* concentrations in canals, rivers, streams, and other water types (e.g., estuaries, runoff, and wastewater) and the second ranked factor associated with *E. coli* concentrations in ponds, reservoirs, and lakes. Season was also the top-ranked factor associated with *Enterococcus* and fecal coliform concentrations in all water types and the second highest-ranked factor associated with total coliform concentrations (Table S7 and S8). After season, state was the second highest-ranked factor associated with *E. coli* concentrations in canals, rivers, streams, and other water types, *Enterococcus* concentrations, and fecal coliform concentrations in ponds, reservoirs, lakes, rivers, and streams (Table S7 and S8). State was the highest-ranked factor associated with *E. coli* concentrations in ponds, reservoirs, and lakes. Freshwater status and the mid-scale and/or coarse-scale methods factors were among the lowest ranked factors for all fecal indicator bacteria, regardless of water type. After accounting for methodological, waterway, and site-specific signals, the top-ranked factors associated with *Enterococcus* concentrations were ecoregion, water type, and hydrologic region. The top-ranked factors associated with total coliform concentration were state, terrestrial habitat type, and ecoregion (Table S8). Ecoregion was strongly associated with *E. coli* concentrations, and habitat type was strongly associated with fecal coliform concentrations in all water types considered.

## DISCUSSION

Our analyses demonstrated water environments are intrinsically complex, and the use of different laboratory and sampling methods limited our ability to untangle this complexity, generating non-comparable results. Data collection and management need to be standardized across studies, and a minimum set of attributes that all studies should collect needs to be established (Table 2). While data from 2,429,990 samples were analyzed, these represent only 77% of the available data because 781,264 samples were discarded due to data quality issues. Even within a single organization, some data could be retained, while some were discarded due to inconsistencies in data collection, cleaning, and management within an organization. Furthermore, physiochemical water attributes and meteorological data, which are often considered during risk assessments for water quality, were not included in the present study due to inconsistencies in data collection and data quality issues. This highlights the need for (i) standardized data collection, cleaning, and management protocols within organizations, and (ii) reporting and archiving water quality data in a consistent way even when studies are conducted by unaffiliated organizations. Similar calls for uniform data standards have been made in other fields, and meeting such standards has become a requirement of certain funding agencies [e.g., US Health and Human Services Office of Minority Health Resource Center (149), US Office of Management and Budget (150), and US Centers for Disease Control and Prevention (151)]. Funding source-generated mandates show that method standardization and data reporting requirements are possible and provide a blueprint for implementing similar standards for water quality data. Such standards could be established by a consortium of key stakeholders, including funding agencies and/or organizations from academia, industry, and government that generate large volumes of water data. Table 2 was developed using lessons learned from this study to help jumpstart the standardization conversation among microbial water quality researchers and end users. The table provides a brief overview of best practices and key considerations for collecting, recording, and reporting of microbial water quality data; Table 2 also cites resources that can be referred to for additional or more in-depth guidance.

Methodological differences between studies indicates findings are not comparable, limiting our ability to identify the broader ecological phenomenon driving foodborne pathogen contamination of waters and complicating the development of effective strategies for managing public health risks associated with contamination. Based on variance partitioning analysis, the impact of methodological differences on observed microbial water quality could not be disentangled from the impact of non-methodological (e.g., region, waterway, and water type) differences when the outcome was the detection of foodborne pathogens, indicator organisms, and fecal indicators. Conditional forest analysis showed that methodological differences were strongly associated with and predictive of observed water quality regardless of site-specific signals from the data. This is consistent with previous studies where the likelihood of detecting foodborne pathogens was strongly associated with the methods used to collect or test samples (15, 16, 27–29, 32). Past studies found that sample type, sample filtration method, and sample volume were strongly associated with the detection of *Listeria* spp., *L. monocytogenes*, *Salmonella*, and/or pathogenic *E. coli* (15, 16, 27–29, 32). Compared to *L. monocytogenes*, *Salmonella* and pathogenic *E. coli* are much more likely to be detected in 10-L grab samples filtered through modified Moore swabs compared to Moore swabs (15, 16, 27, 28). Our analysis also found significant differences in the likelihood of foodborne pathogen detection by filtration method but did not observe a significant difference in *L. monocytogenes* detection between membrane-filtered and modified Moore swab filtered samples, unlike a previous study (15). This difference may be due to substantially fewer samples (*N* = 29) in the previous study than the present study (*N* = 5,442) and most studies that tested for *L. monocytogenes* used the same or similar laboratory methods.

The impact of methodology on foodborne pathogen detection may be confounded by the heterogeneity of methods and the fact that some methods were only used by a single or small number of studies all conducted in a single region and/or on a single water type. For example, all grab samples that were not filtered and were tested for *Salmonella* came from a single study conducted in Sinaloa, Mexico. Therefore, we do not know if some of the larger odds ratios reported in Table S5 are due to actual methodological differences or confounding between methodology, region, study, laboratory, or water type. The size and heterogeneity of the data set reported here help reduce the impact of this confounding for commonly used methods (e.g., membrane filtration, modified Moore swabs, and Moore swabs).

With the exception of *Listeria* spp. and *L. monocytogenes*, larger sample volumes were associated with an increased likelihood of foodborne pathogen detection. Past studies demonstrating a similar trend have hypothesized that the inverse relationship between *Listeria* recovery and sample volume could be due to more competitive microflora in larger volume samples (15, 16). Regardless of why *Listeria* detection was inversely related to volume, *Salmonella* and pathogenic *E. coli* detection were positively associated with sample volume. This is consistent with a past study that found a 26-fold and 44-fold increase in odds of *Salmonella* recovery from 10-L grab samples compared to 1.0 and 0.1 L, respectively (32). A plateau effect was seen in the relationship between sample volume and odds of *Salmonella* and pathogenic *E. coli* detection; contrary to this previous study, increasing volume above 1 L does not substantially increase the odds of detection. Since collecting and processing larger volumes of water are more labor and capital intensive than collecting and processing smaller volumes, knowing the threshold for diminishing returns for sample volume is critical.

Similar to sample type, filtration method, sample volume, and pathogen detection method were all strongly associated with *Salmonella* and pathogenic *E. coli* detection. While detection method was not significantly associated with *L. monocytogenes* detection, this may be an artifact of the fact that only one study representing 36 samples from a single water type and region used a molecular approach to detect *L. monocytogenes* (16). A previous study found *Listeria* detection was much more frequent when a TaqMan assay was used compared to a culture-based method (23). In the present study, odds of *Salmonella* detection were much lower, and odds of pathogenic *E. coli* detection were much higher when molecular-based as opposed to culture-based detection was used. A strong association between pathogenic *E. coli*, STEC, and EPEC detection and detection method is unsurprising, given the difficulties associated with culture-based detection of these foodborne pathogens (152–157). The finding that the odds of *Salmonella* detection were negatively associated with the use of molecular detection methods may be due to the coarse classification as either a culture-based or a molecular method. This binary classification ignored many of the substantial differences between protocols within these broad categories. For example, some studies used a culture-based methods that included PCR confirmation following isolation or a PCR-screen prior to isolation, while others used culture-based methods and did not include any form of molecular confirmation. The target gene was significantly associated with both likelihood of *Salmonella* and pathogenic *E. coli* detection in the present and previous studies (158–160). Similarly, the media used, incubation temperature, and timing are all known to affect foodborne pathogen recovery by culture-based methods (154, 156, 161, 162), and these were not considered here. Breaking down detection methods into culture and molecular-based approaches may have made it difficult to see the distinctions between various culture-based methods. This could have made it seem like there were fewer differences between culture-based and molecular approaches than there were. Despite this limitation, the findings of this and previous studies (16, 23, 158–160) highlight the impact of methodological differences between studies on observed water quality and suggest results may not be directly comparable between studies that used culture- and molecular-based methods. It is important to note that the difference in likelihood of detection by method could be due to higher false positive rates for culture-based or false negative rates for molecular-based methods.

Methodological factors were less impactful in the fecal indicator conditional forest analyses than non-methodological factors. This is unsurprising as many enumeration methods are considered equivalent to EPA Method 1603 (136, 137). Thus, existing fecal indicator data may be better suited for meta-analysis and use in large-scale modeling efforts than foodborne pathogen data. Much of the fecal indicator data (99.9% of *E. coli*, 86.3% of *Enterococcus*, and 100% of fecal and total coliform data) reported here were generated using culture-based as opposed to molecular methods; thus, differences due to the use of molecular methods could not be fully explored. Previously, higher *E. coli* concentrations using PCR-based as opposed to culture-based enumeration methods have been reported (163). While combining fecal indicator data enumerated using molecular and culture-based approaches will require further consideration, the level of methods standardization recommended for foodborne pathogen detection may not be needed for routine fecal indicator monitoring. There is still a need for standardization in data reporting and management since the majority of the 781,264 samples discarded for data issues were for fecal indicators.

Opportunities for site or waterway-specific management exist since substantial variance in microbial water quality was still uniquely attributable to non-methodological factors even though methodological signals could not be disentangled from non-methodological signals. While little to no variance in microbial water quality was uniquely attributable to methodological factors, a substantial amount of variance was uniquely attributable to non-methodological factors. More variance was uniquely attributable to sampling site and waterway compared to other non-methodological factors (e.g., region, year, and season), highlighting the dependency of microbial water quality on local environmental context. This is consistent with past studies that found evidence of strong site and waterway effects, and/or concluded that microbial water quality was dependent on the local environmental context (16, 164, 165). Such dependency on local environmental factors complicates the establishment of one-size-fits-all water quality standards or universal best practices for the use of water.

While microbial water quality is strongly affected by local environmental factors, we implemented conditional forest analysis to see if and which regional, temporal, and water type factors were most strongly associated with each microbial water quality target. Water type-related factors were the top-ranked feature for five of seven foodborne pathogen forests, although the exact factor varied by microbial target. Conceptually, this is logical because different water types represent distinct environments where different processes drive water quality. For example, non-tidal rivers and streams are unidirectional and strongly influenced by upstream environmental conditions, but the impact of upstream conditions is reduced for other water types, such as the Great Lakes or seeps fed by groundwater. Past studies that sampled multiple water types often reported drastically different foodborne pathogen prevalence for the sampled water types (7, 12, 40, 166).

Every forest that considered regional factors in the present study included a regional feature among the top 10% of factors. Environmentally derived factors based on ecoregion (*N* = 9), habitat type (terrestrial = 6, aquatic = 2), climate (*N* = 1), or hydrologic region (*N* = 1) were the top-ranked regional features for 14 of the 17 forests. This is consistent with past studies that have repeatedly associated microbial water quality with environmental parameters, including weather and hydrologic characteristics (8–11, 167–172). Here, region and water type were strongly associated with several of the microbial water quality parameters considered in the present study.

### Conclusion

This analysis shows that our current understanding of foodborne pathogen dynamics in water systems is limited by methodological confounders and brings into question the comparability of foodborne pathogen data generated by studies using different sampling and/or laboratory methods. Foodborne pathogen ecology in water is complex, without the added complications of methodological differences. Without comparability, it is difficult to identify the broader ecological phenomenon driving foodborne pathogen contamination of waters and complicating the development of effective strategies for managing public health risks associated with microbial contamination. This study highlights the need for standardizing sampling and laboratory methods used for microbial water quality testing. Future work could include comparing methods for equivalency. Similar standards are needed for data collection, management, and reporting. Since there is a diversity of methods used for a variety of sample types and fields within applied microbiology, similar analyses are needed to ensure the comparability of findings for other subfields of food and environmental microbiology. If methodological and data standards are not implemented, comparability will continue to be an issue, and there will always be a caveat to future findings.

## MATERIALS AND METHODS

### Data sets

Water quality data from peer-reviewed papers, publicly available databases, citizen science groups, and government programs were compiled (Table 1; database: https://github.com/wellerd2/Weller-et-al-2024-AEM-Datasets/tree/main). For each sampling site, water type, freshwater/saltwater status, and waterway name were determined; if these data were not available, Google Earth and Google Maps were used to obtain it. Four different, nested classifications for water type were used. The finest scale variable (fine-scale water type) included 27 categories; mid-scale water type included 20 categories; coarse-scale water type included 10 categories; and general-scale water type included four categories. For example, urban ponds (fine-scale water type) collapse into ponds (mid-scale), which collapse into lakes/ponds/reservoirs (coarse-scale), which collapse into surface water (general). Footnote *h* in Table S9 describes all categories of the nested water type classification scheme used here.

Sampling and laboratory methods for each study were previously published, available online, or shared by the data set owners through personal correspondence. Key methods data were extracted and added to sample attributes. If methods data were not available for a sample, the sample was excluded from the study. Sample type, detection method, sample volume, and filtration method were recorded for samples with foodborne pathogen data. Since several of the analytical methods used here could not handle missing data, Moore swab samples were assigned a volume of 10 L, which is the largest grab sample volume collected in this study.

Studies often tested samples for a microbial target using a culture-based method and confirmed presumptive positives using a molecular method or vice versa. However, most of the data sets that included a confirmation step only provided data on if both the culture and molecular results were positive (e.g., included a single column for if it was culture-positive and molecular confirmed, not separate columns for culture and molecular results). As a result, only a single positive-negative designation was reported for most data sets. Thus, a sample was classified as being tested using a culture-based method, if there was any culture-based step (i.e., culture-based detection with no confirmation, molecular detection with culture confirmation, and culture-based detection with molecular confirmation). However, if results were available for both the culture and molecular tests separately, we included these as separate data rows with a common sample site ID and date; indeed, if any sample was tested by multiple methods (e.g., different filtration methods and sample volumes), then these results were treated as separate data rows linked by site ID and date. If a sample was screened by molecular detection with culture confirmation, it was considered molecular. If molecular methods were used, target genes were noted. Since there are multiple types of pathogenic *Escherichia coli*, samples were categorized as positive-negative for any pathogenic *E. coli*, and for EPEC (based on detection of the *eaeA* gene), STEC (based on detection of the *stx* genes), and *E. coli* O157 (using culture-based methods and PCR confirmation).

Fecal indicator methods were classified by the protocol used for enumeration and separately by the media used. Since not all studies used an established protocol, three different approaches were used to categorize methods (Table S1). A fine-scale method factor was created and reflects the established protocol used (e.g., Standard Method 9222B) or if a specific protocol could not be identified, study ID was used in place of the fine-scale method. A mid-level method factor was used to capture methods that were almost the same but with slight variations (e.g., Standard Method 9222B and Standard Method 9222C were Standard Method 9222). A coarse-level factor was used to capture if direct plating, membrane filtration, most probable number estimation, or a molecular approach was used for enumeration.

### Assigning samples to regions

Using GPS coordinates and county, we classified samples separately into regions using different regional schemes. Different approaches for grouping samples into regions were used to determine if there was a regional scheme that accounted for the greatest variance for each microbial target. Fourteen regional schemes were considered, including schemes based on the biome, ecoregion, aquatic and terrestrial habitat type, climate region, hydrologic region (based on USGS HUC2 unit codes; 173) regional classifications used by US federal agencies (i.e., Census Bureau and US Environmental Protection Agency), and on agricultural practices and/or output (USDA-Farm Resource Regions, Farm Production Regions, National Agricultural Statistics Service Regions, and Human Geography Agricultural Regions). For US-specific schemes, sites outside the US were assigned to the region of the closest US site.

### Statistical analysis

All analyses were performed in R version 4.0.3 (R Foundation for Statistical Computing, Vienna, Austria). A summary of all analyses implemented here, as well as their reason for being implemented and key model specifications, is outlined in Table 3 . Table S9 lists methodological and non-methodological factors used here.

**TABLE 3:** Summary of statistical analysis performed, the goal of each analysis, R packages and model specifications needed for each analysis, and the data subsets that analysis was performed on

Analysis	Goal	R packages used	Model specifications	Data subset analysis were performed on
Conditional forest analysis using methodological, spatial, and temporal factors	To characterize hierarchical associations between methodological, spatial, and temporal factors	*lme4*, *moreparty*	Prior to training each forest, a general linear model or generalized linear model was fit with random effect of site ID nested in waterway, and residuals from this model were the dependent variable in the forest.	*Salmonella*. *Listeria* spp. *L. monocytogenes*. Pathogenic *E. coli*. EPEC. STEC. *E. coli* O157. Generic *E. coli* by water type. *Enterococcus*. Total coliforms. Fecal coliforms by water type.
Conditional forest analysis using water type and regional factors	To determine if water type and/or regional scheme was more strongly associated with each microbial target after accounting for other confounding factors (e.g., methodological).	*lme4*, *moreparty*	Prior to training each forest, a general linear model or generalized linear model was fit with random effects for each methodological variable, and residuals from this model were the dependent variable in the forest.	
Variance partitioning analysis	To quantify the variance in likelihood of target detection that was uniquely versus jointly attributable to methodological versus non-methodological factors	*vegan*	Four sets of analyses were performed using the following matrices, each consisting of one or more factors: A methodological matrix and a non-methodological matrix. A methodological matrix and three non-methodological matrices (state and region, waterway/site and water type, and temporal). A methodological matrix and three non-methodological matrices (waterway/site, water type, and temporal). A methodological matrix and two non-methodological matrices (sampling site and all other non-methodological factors).	*Salmonella.* *Listeria* spp. *L.monocytogenes.* Pathogenic *E. coli.* EPEC. STEC.
Generalized linear models	To quantify how using a given method influenced the likelihood of detecting a microbial target	*lme4*	Mixed model, binomial family, and logit link. Random effects of site nested in waterway nested in state, and of season. Fixed effect for the methodological factor of interest. Benjamin-Hochberg multiple comparison correction applied.	*Salmonella* *Listeria* spp. *L. monocytogenes*. Pathogenic *E. coli*. EPEC. STEC.
Tukey’s HSD^*a*^	To determine if specific methodological choices generated comparable data (e.g., if there was a significant difference in the likelihood of detection if membrane filtration, modified Moore swabs, or no filter was used).	*multcomp*	Tukey’s HSD was applied post hoc to the model object returned from the generalized linear models run above when the methodological factor of interest was categorical.	

^*a*^ HSD, honestly significant difference.

### Conditional forest analysis

Conditional forest analysis was used to characterize hierarchical associations between methodological, spatial, and temporal factors to better understand if and when methodological differences affected observed microbial water quality, and if interactions between methods and other factors might affect observed microbial water quality. Due to the large number of samples with *E. coli* or fecal coliform data, we lacked the computational resources to implement a single forest for either fecal indicator. Instead, separate *E. coli* and fecal coliform forests were implemented for samples collected from canals, lakes and ponds, streams, rivers, and all other water types (e.g., groundwater and ocean). As a result, five separate forests were run using the *E. coli* and fecal coliform data. Prior to training each forest, a general linear model (for continuous outcomes) or generalized linear model (for binary outcomes) with a random effect of site ID nested in waterway was fit using the *lme4* package (174); the dependent variable in the forest was the residuals from this model.

Separately, conditional forest analysis was used to determine if water type and/or regional scheme was more strongly associated with each microbial target after accounting for other confounding factors (e.g., methodological factors). As described above, separate water type-specific models were implemented for the *E. coli* and fecal coliform data. Prior to training each forest, a general linear model or generalized linear model was fit with random effects for each methodological factor available for the microbial target using the *lme4* package (174). If the model failed to converge or had singular fit, the model was re-parameterized (e.g., random effect shifted to fixed effect, or a factor was dropped); the dependent variable in the forest was the residuals from this model. For both sets of forests, unbiased conditional forest analysis was implemented using the *moreparty* package (175). Conditional variable importance was calculated to identify factors in each forest that were most strongly associated with each microbial target; conditional variable importance was used because it is unbiased by correlation between covariates.

### Variance attributable to methodological versus non-methodological signals

Variance partitioning analysis was implemented using the *vegan* package (176) to quantify the variance in likelihood of foodborne pathogen detection uniquely and jointly attributable to methodological and non-methodological factors. For foodborne pathogen targets, four sets of variance partitioning analyses were performed using the following sets of matrices (i) state and region, site (waterway, site, water type, and freshwater status), temporal (season and year), and methodological factors; (ii) waterway (waterway and sampling site), water type (water type and freshwater status), temporal and methodological factors; (iii) methodological versus all other non-methodological factors; and (iv) sampling site, methodological factors, and all other non-methodological factors. Due to the computational intensity of these analyses and the large number of samples, variance partitioning analysis was not performed for fecal indicator bacteria.

### Mixed models were implemented to identify “comparable” methods

To quantify how using a given sample processing or laboratory method influenced the likelihood of foodborne pathogen or indicator organism detection, generalized linear models were implemented using the *lme4* package (174). The models were implemented with the binomial family, a logit link, random effects of site nested in waterway nested in state, a random effect of season, and a fixed effect for the methodological factor of interest. To determine if specific methodological choices generated comparable data (e.g., if there was a significant difference in the likelihood of detection if membrane filtration, modified Moore swabs, or no filter was used), Tukey’s honestly significant difference was performed using the *multcomp* package (177). To account for multiple comparisons, the Benjamin-Hochberg multiple comparison correction was used. These analyses were only performed using the foodborne pathogen data (as opposed to the fecal indicator data) because (i) foodborne pathogen contamination is the primary outcome of interest and (ii) methodological factors were consistently among the top-ranked factors in the foodborne pathogen forests but not in fecal indicator forests.

## Data Availability

Table 1 outlines the source of all data used in this study including all original study authors/data owners. Datasets and locations are available at: https://github.com/wellerd2/Weller-et-al-2024-AEM-Datasets/tree/main, or by contacting the study authors (L.S., C.M., D.W.) for confidential data. Confidential data including GPS coordinates, site names, and waterway names were dropped at request of the original study authors due to commercial agricultural farm privacy (also prior published studies have kept this information confidential), but is available upon request of the original study authors (listed in Table 1).

## AFTER EPUB

*[This article was published on 12 January 2024 with an inaccuracy in Fig. 1. The figure was updated in the current version, posted on 18 January 2024.]*

## SUPPLEMENTAL MATERIAL

Figure S1, Tables S1 to S9 aem.01835-23-s0001.docx Supplemental materials providing additional details or outputs of data analyses.

ASM does not own the copyrights to Supplemental Material that may be linked to, or accessed through, an article. The authors have granted ASM a non-exclusive, world-wide license to publish the Supplemental Material files. Please contact the corresponding author directly for reuse.
