# Supplementary material for: Methodological differences between studies confound one-size-fits-all approaches to managing surface waterways for food and water safety
Source: Appl Environ Microbiol. 2024 Jan 12;90(2):e01835-23. doi: 10.1128/aem.01835-23 (PMC10880618; doi:10.1128/aem.01835-23)
Supplement: Figure S1, Tables S1 to S9 — Supplemental materials providing additional details or outputs of data analyses. [file aem.01835-23-s0001.docx]

Supplemental Figure 1: Location and water type for sampling sites in Canada, the continental United States, and Mexico. For legibility, wetlands are not shown and GPS coordinates were jittered slightly. (The map was created in R using the ggplot2 and sf packages.)


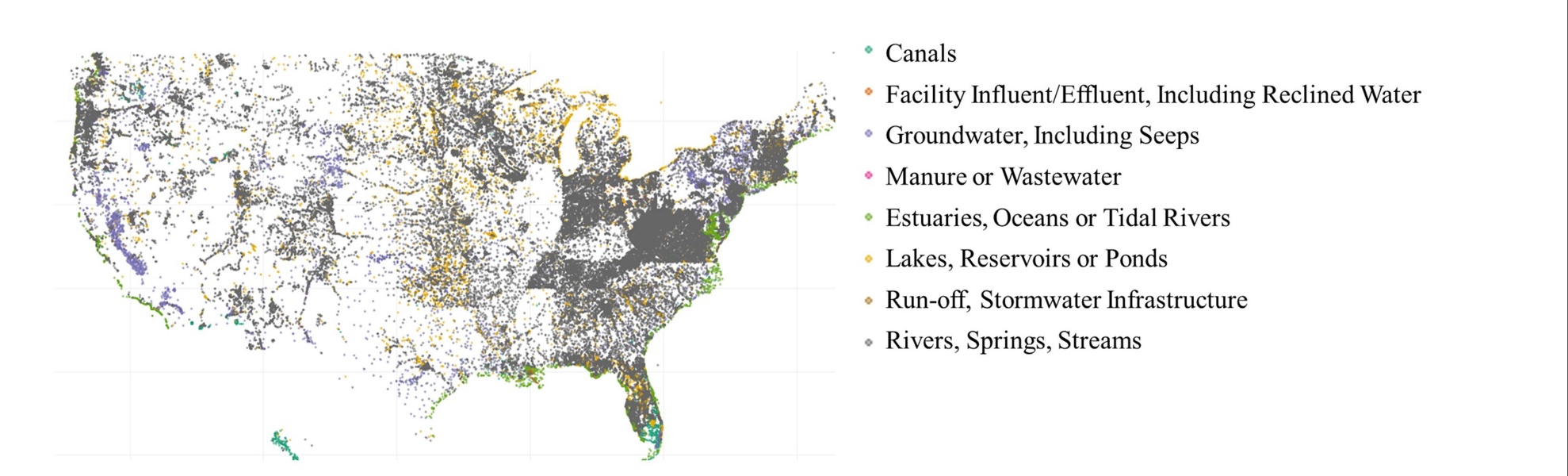


Supplemental Table 1: Summary of enumeration methods used to quantify *E. coli, Enterococcus*, fecal coliform and total coliform levels in the datasets compiled by the present study. Some studies did not use a standard protocol (e.g., EPA Method 1603, Standard Method 1922D), and instead used a method unique to their project.^a^

| **Target** | **General Method** | **Specific Method** | **No. of Samples** |
| --- | --- | --- | --- |
| *E. coli* | | |  |
|  | Direct Plating | |  |
|  |  | Coliscan Easygel | 10,752 |
|  |  | EPA Method 1604 | 24,567 |
|  |  | Petrifilm | 441 |
|  |  | Study-Specific Methods ^a^ | 12,825 |
|  | Membrane Filtration | |  |
|  |  | EPA Method 1103 | 133,633 |
|  |  | EPA Method 1105 | 12 |
|  |  | EPA Method 1603 | 106,500 |
|  |  | FDA's Bacteriological Analytical Manual (BAM) | 390 |
|  |  | m-ColiBlue | 16,481 |
|  |  | Standard Method 9213D | 87,992 |
|  |  | Standard Method 9222G | 77,140 |
|  |  | Study-Specific Methods ^a^ | 281 |
|  | Most Probable Number | |  |
|  |  | EPA Method 1104 | 723 |
|  |  | Hach 10018 | 314 |
|  |  | IDEXX Colilert Quanti-Tray | 904,814 |
|  |  | Standard Method 9221F | 12,555 |
|  |  | Study-Specific Methods ^a^ | 513 |
|  | Molecular/QPCR | |  |
|  |  | Study-Specific Methods ^a^ | 1,464 |
| *Enterococcus* | | |  |
|  | Membrane Filtration | |  |
|  |  | EPA Method 1106 | 142 |
|  |  | EPA Method 1600 | 36,097 |
|  |  | Standard Method 9230C | 29,581 |
|  |  | Study-Specific Methods ^a^ | 635 |
|  | Most Probable Number | |  |
|  |  | IDEXX Enterolert Quanti-Tray | 60,313 |
|  |  | Standard Method 9230B | 1,020 |
|  |  | Standard Method 9230D | 2,532 |
|  |  | Study-Specific Methods ^a^ | 528 |
|  | Molecular/QPCR | |  |
|  |  | EPA Method 1606 | 5542 |
|  |  | EPA Method 1609 | 195 |
|  |  | Study-Specific Methods ^a^ | 14,993 |
| Fecal Coliform | | |  |
|  | Direct Plating | |  |
|  |  | Petrifilm | 221 |
|  |  | Study-Specific Methods ^a^ | 22 |
|  | Membrane Filtration | |  |
|  |  | FDA's Bacteriological Analytical Manual (BAM) | 118 |
|  |  | Standard Method 9222D | 610324 |
|  |  | Standard Method 9222E | 10452 |
|  |  | Standard Method 9222, Other ^b^ | 21,914 |
|  |  | Study-Specific Methods ^a^ | 7,315 |
|  | Most Probable Number | |  |
|  |  | AOAC Method 978.23 | 249,272 |
|  |  | APHA 3.2B | 106,737 |
|  |  | Hach 8001 | 8,460 |
|  |  | IDEX Quanti-Tray | 14,614 |
|  |  | Standard Method 9221E | 61,743 |
|  |  | USGS Method B-0051-85 | 22,578 |
|  |  | Study-Specific Methods ^a^ | 21,430 |
| Total Coliforms | | |  |
|  | Direct Plating | |  |
|  |  | Coliscan Easygel | 27 |
|  |  | EPA Method 1604 | 6,794 |
|  |  | Study-Specific Methods ^a^ | 6,741 |
|  | Membrane Filtration | |  |
|  |  | EPA Method 9132 | 1,749 |
|  |  | FDA's Bacteriological Analytical Manual (BAM) | 393 |
|  |  | m-ColiBlue | 1,824 |
|  |  | Standard Method 9222B | 29,382 |
|  |  | USGS Method B-0025-85 | 1,704 |
|  |  | Study-Specific Methods ^a^ | 8,068 |
|  | Most Probable Number | |  |
|  |  | IDEXX Colilert Quanti-Tray | 109374 |
|  |  | Standard Method 9221BC | 8556 |
|  |  | USGS Method B-0040-85 | 4 |
|  |  | Study-Specific Methods ^a^ | 10,943 |

^a^ For legibility all samples tested using a study specific method are aggregated in the present table under “Study-Specific Method”. However, unique identifiers were assigned to each unique method and used as separate levels of the specific method variable included in the forest analyses. For example, six studies used an unique method for *E. coli*enumeration and were each assigned a unique protocol identified (i.e., Specific Methods A, B, C, D, F, G).

**^b^** Four USGS water quality monitoring programs used a protocol similar to one of the Standard Method 9222 protocols for fecal coliform enumeration. However, the exact protocol was not named, and instead were briefly described as Bac32, Bac33, Bac35, and Bac93 (<https://help.waterdata.usgs.gov/codes-and-parameters/code/method_cd_query?fmt=html>). While we aggregated these methods together in the table here, unique identifiers were assigned to each method and used as separate levels of the specific method variable included in the forest analyses.

**Supplemental Table 2:** Summary of sampling, sample processing and laboratory methods used to quantify *Salmonella*, *Listeria* spp., *L. monocytogenes*, and pathogenic *E. coli* by the datasets compiled by present study.

| **Dataset** | | **Sample Type** | **Filter Type ^a^** |  | ***Salmonella*** | | | |  | | ***Listeria* spp.** | | | |  | ***L. monocytogenes*** | | | |  | **Pathogenic *E. coli*** | | | |
| --- | --- | --- | --- | --- | --- | --- | --- | --- | --- | --- | --- | --- | --- | --- | --- | --- | --- | --- | --- | --- | --- | --- | --- | --- |
|  |  |  |  |  | **Vol. ^b^** | **Detection Method ^c^** | **Samples ^d^** | |  | **Vol. ^b^** | | **Detection Method ^c^** | **Samples ^d^** | |  | **Vol. ^b^** | **Detection Method ^c^** | **Samples ^d^** | |  | **Vol. ^b^** | **Detection Method ^c^** | **Samples ^d^** | |
|  |  |  |  |  |  |  | **Pos.** | **Tot.** |  |  |  |  | **Pos.** | **Tot.** |  |  |  | **Pos.** | **Tot.** |  |  |  | **Pos.** | **Tot.** |
| ANF | |  |  |  |  |  |  |  |  |  | |  |  |  |  |  |  |  |  |  |  |  |  |  |
|  | Canal | Grab | None |  | 25 | Cx | 51 | 345 |  | ^d^ | |  |  |  |  |  |  |  |  |  | 3.33 | Cx | 17 | 351 |
|  | Lake or Reservoir | Grab | None |  | 25 | Cx | 1 | 14 |  |  | |  |  |  |  |  |  |  |  |  | 3.33 | Cx | 0 | 14 |
|  | Pond | Grab | None |  | 25 | Cx | 2 | 12 |  |  | |  |  |  |  |  |  |  |  |  | 3.33 | Cx | 0 | 12 |
|  | River | Grab | None |  | 25 | Cx | 6 | 23 |  |  | |  |  |  |  |  |  |  |  |  | 3.33 | Cx | 3 | 23 |
|  | Stream | Grab | None |  | 25 | Cx | 1 | 3 |  |  | |  |  |  |  |  |  |  |  |  | 3.33 | Cx | 0 | 3 |
| BCB | |  |  |  |  |  |  |  |  |  | |  |  |  |  |  |  |  |  |  |  |  |  |  |
|  | Stream | Grab | MF |  | 50 | Cx | 9 | 30 |  |  | |  |  |  |  |  |  |  |  |  | 50 | Cx | 26 | 30 |
| CEG | |  |  |  |  |  |  |  |  |  | |  |  |  |  |  |  |  |  |  |  |  |  |  |
|  | Stream | Grab | MF |  | 50 | CIDT | 4 | 22 |  |  | |  |  |  |  |  |  |  |  |  | 50 | CIDT | 21 | 22 |
| CNP | |  |  |  |  |  |  |  |  |  | |  |  |  |  |  |  |  |  |  |  |  |  |  |
|  | Lake or Reservoir |  |  |  |  |  |  |  |  |  | |  |  |  |  |  |  |  |  |  |  |  |  |  |
|  |  | Grab | MF |  |  |  |  |  |  |  | |  |  |  |  |  |  |  |  |  | 50 | CIDT | 0 | 26 |
|  |  | Grab | MF |  |  |  |  |  |  |  | |  |  |  |  |  |  |  |  |  | 100 | CIDT | 0 | 18 |
|  | River |  |  |  |  |  |  |  |  |  | |  |  |  |  |  |  |  |  |  |  |  |  |  |
|  |  | Grab | MF |  |  |  |  |  |  |  | |  |  |  |  |  |  |  |  |  | 50 | CIDT | 0 | 8 |
|  |  | Grab | MF |  |  |  |  |  |  |  | |  |  |  |  |  |  |  |  |  | 100 | CIDT | 2 | 78 |
|  | Stream |  |  |  |  |  |  |  |  |  | |  |  |  |  |  |  |  |  |  |  |  |  |  |
|  |  | Grab | MF |  |  |  |  |  |  |  | |  |  |  |  |  |  |  |  |  | 20 | CIDT | 0 | 2 |
|  |  | Grab | MF |  |  |  |  |  |  |  | |  |  |  |  |  |  |  |  |  | 50 | CIDT | 4 | 148 |
|  |  | Grab | MF |  |  |  |  |  |  |  | |  |  |  |  |  |  |  |  |  | 100 | CIDT | 24 | 302 |
| CLY | |  |  |  |  |  |  |  |  |  | |  |  |  |  |  |  |  |  |  |  |  |  |  |
|  | River | Moore Swab | - |  |  | CIDT | 8 | 9 |  |  | |  |  |  |  |  | CIDT | 4 | 9 |  |  | CIDT | 9 | 9 |
|  | Stream | Moore Swab | - |  |  | CIDT | 22 | 27 |  |  | |  |  |  |  |  | CIDT | 17 | 27 |  |  | CIDT | 21 | 27 |
| CSH | |  |  |  |  |  |  |  |  |  | |  |  |  |  |  |  |  |  |  |  |  |  |  |
|  | Pond | Grab | None |  | 1830 | Cx | 22 | 48 |  |  | |  |  |  |  |  |  |  |  |  |  |  |  |  |
|  | Runoff | Grab | None |  | 1830 | Cx | 18 | 47 |  |  | |  |  |  |  |  |  |  |  |  |  |  |  |  |
|  | Stream | Grab | None |  | 1830 | Cx | 12 | 12 |  |  | |  |  |  |  |  |  |  |  |  |  |  |  |  |
| FSL | |  |  |  |  |  |  |  |  |  | |  |  |  |  |  |  |  |  |  |  |  |  |  |
|  | Canal | Grab | MF |  |  |  |  |  |  | 250 | | Cx | 0 | 1 |  | 250 | Cx | 0 | 1 |  | 250 | Cx | 2 | 18 |
|  | Ditch | Grab | MF |  | 250 | Cx | 1 | 48 |  | 250 | | Cx | 41 | 109 |  | 250 | Cx | 12 | 109 |  |  |  |  |  |
|  | Estuary or Tidal River | Grab | MF |  |  |  |  |  |  | 250 | | Cx | 9 | 17 |  | 250 | Cx | 1 | 17 |  | 250 | Cx | 0 | 14 |
|  | Lake or Reservoir ^f^ | Grab | MF |  | 250 | Cx | 0 | 14 |  | 250 | | Cx | 21 | 112 |  | 250 | Cx | 4 | 112 |  |  |  |  |  |
|  | Ocean | Grab | MF |  |  |  |  |  |  | 250 | | Cx | 0 | 5 |  | 250 | Cx | 0 | 5 |  | 250 | Cx | 0 | 20 |
|  | Pond ^g^ | Grab | MF |  | 250 | Cx | 1 | 20 |  | 250 | | Cx | 74 | 204 |  | 250 | Cx | 50 | 204 |  | 250 | Cx | 0 | 8 |
|  | River | Grab | MF |  | 250 | Cx | 0 | 8 |  | 250 | | Cx | 13 | 41 |  | 250 | Cx | 2 | 41 |  | 250 | Cx | 3 | 201 |
|  | Stream | Grab | MF |  | 250 | Cx | 19 | 201 |  | 250 | | Cx | 188 | 447 |  | 250 | Cx | 96 | 447 |  |  |  |  |  |
| FUR | |  |  |  |  |  |  |  |  |  | |  |  |  |  |  |  |  |  |  |  |  |  |  |
|  | Lake or Reservoir | Grab | MF |  |  |  |  |  |  |  | |  |  |  |  |  |  |  |  |  | 100 | Cx | 0 | 1 |
|  | River | Grab | MF |  |  |  |  |  |  |  | |  |  |  |  |  |  |  |  |  | 100 | Cx | 12 | 12 |
|  | Stream | Grab | MF |  |  |  |  |  |  |  | |  |  |  |  |  |  |  |  |  | 100 | Cx | 2 | 5 |
| GUF | |  |  |  |  |  |  |  |  |  | |  |  |  |  |  |  |  |  |  |  |  |  |  |
|  | Pond | Grab | None |  | 2440 | Cx | 82 | 429 |  |  | |  |  |  |  | 500 | Cx | 15 | 48 |  |  |  |  |  |
| LCM | |  |  |  |  |  |  |  |  |  | |  |  |  |  |  |  |  |  |  |  |  |  |  |
|  | Ground-water | Grab | MF |  |  |  |  |  |  |  | |  |  |  |  |  |  |  |  |  | 100 | CIDT | 8 | 16 |
|  | Manure or  Wastewater | Grab | MF |  |  |  |  |  |  |  | |  |  |  |  |  |  |  |  |  | 100 | CIDT | 10 | 10 |
|  | Pond | Grab | MF |  |  |  |  |  |  |  | |  |  |  |  |  |  |  |  |  | 100 | CIDT | 12 | 18 |
|  | River | Grab | MF |  |  |  |  |  |  |  | |  |  |  |  |  |  |  |  |  | 100 | CIDT | 24 | 27 |
| LTH | |  |  |  |  |  |  |  |  |  | |  |  |  |  |  |  |  |  |  |  |  |  |  |
|  | Lake or Reservoir | Grab | MF |  | 500 | CIDT | 39 | 168 |  | 500 | | Cx | 60 | 168 |  | 500 | Cx | 104 | 168 |  | 500 | CIDT | 5 | 168 |
|  | River | Grab | MF |  | 500 | CIDT | 46 | 165 |  | 500 | | Cx | 119 | 165 |  | 500 | Cx | 121 | 165 |  | 500 | CIDT | 19 | 165 |
| LW | |  |  |  |  |  |  |  |  |  | |  |  |  |  |  |  |  |  |  |  |  |  |  |
|  | Pond | Grab | MF |  | 250 | Cx | 17 | 56 |  | 250 | | Cx | 8 | 56 |  | 250 | Cx | 7 | 56 |  |  |  |  |  |
|  | River | Grab | MF |  | 250 | Cx | 1 | 20 |  | 250 | | Cx | 1 | 20 |  | 250 | Cx | 0 | 20 |  |  |  |  |  |
|  | Stream | Grab | MF |  | 250 | Cx | 8 | 44 |  | 250 | | Cx | 4 | 44 |  | 250 | Cx | 2 | 44 |  |  |  |  |  |
| METG | |  |  |  |  |  |  |  |  |  | |  |  |  |  |  |  |  |  |  |  |  |  |  |
|  | River | Grab | MF |  | 2500 | MG | 1 | 4 |  |  | |  |  |  |  |  |  |  |  |  | 2500 | CIDT | 2 | 4 |
|  | Stream | Grab | MF |  | 600 | MG | 5 | 8 |  |  | |  |  |  |  |  |  |  |  |  |  |  |  |  |
| MIR | |  |  |  |  |  |  |  |  |  | |  |  |  |  |  |  |  |  |  |  |  |  |  |
|  | Pond | Grab | MF |  |  |  |  |  |  |  | |  |  |  |  |  |  |  |  |  | 100 | Cx | 2 | 2 |
|  | River | Grab | MF |  |  |  |  |  |  |  | |  |  |  |  |  |  |  |  |  | 100 | Cx | 42 | 45 |
|  | Stream | Grab | MF |  |  |  |  |  |  |  | |  |  |  |  |  |  |  |  |  | 100 | Cx | 19 | 19 |
| NASA | |  |  |  |  |  |  |  |  |  | |  |  |  |  |  |  |  |  |  |  |  |  |  |
|  | Lake or Reservoir | Moore Swab | - |  |  | Cx | 64 | 131 |  |  | |  |  |  |  |  | Cx | 58 | 129 |  |  | Cx | 6 | 131 |
|  | Pond | Moore Swab | - |  |  | Cx | 46 | 165 |  |  | |  |  |  |  |  | Cx | 71 | 163 |  |  | Cx | 39 | 165 |
|  | River | Moore Swab | - |  |  | Cx | 568 | 982 |  |  | |  |  |  |  |  | Cx | 242 | 959 |  |  | Cx | 115 | 982 |
|  | Stream | Moore Swab | - |  |  | Cx | 1018 | 1701 |  |  | |  |  |  |  |  | Cx | 906 | 1671 |  |  | Cx | 107 | 1828 |
| NUF | |  |  |  |  |  |  |  |  |  | |  |  |  |  |  |  |  |  |  |  |  |  |  |
|  | Lake or Reservoir | Grab | None |  | 33.3 | Cx | 4 | 12 |  |  | |  |  |  |  |  |  |  |  |  |  |  |  |  |
|  | River | Grab | None |  | 33.3 | Cx | 13 | 60 |  |  | |  |  |  |  |  |  |  |  |  |  |  |  |  |
| NWIS | |  |  |  |  |  |  |  |  |  | |  |  |  |  |  |  |  |  |  |  |  |  |  |
|  | Great Lakes | Grab | None |  |  | CIDT | 1 | 59 |  |  | |  |  |  |  |  |  |  |  |  |  |  |  |  |
|  | Ground-water | Grab | None |  |  | CIDT | 5 | 11 |  |  | |  |  |  |  |  |  |  |  |  |  |  |  |  |
|  | River | Grab | None |  |  | CIDT | 49 | 677 |  |  | |  |  |  |  |  |  |  |  |  | 25 | CIDT | 3 | 384 |
|  | Stream | Grab | None |  |  | CIDT | 21 | 212 |  |  | |  |  |  |  |  |  |  |  |  | 25 | CIDT | 3 | 168 |
| PAWQ | |  |  |  |  |  |  |  |  |  | |  |  |  |  |  |  |  |  |  |  |  |  |  |
|  | Canal | Grab | MMS |  | 10000 | Cx | 107 | 279 |  | 10000 | | Cx | 3 | 110 |  | 10000 | Cx | 3 | 110 |  | 10000 | Cx | 116 | 283 |
|  | River | Grab | MMS |  | 10000 | Cx | 5 | 9 |  |  | |  |  |  |  |  |  |  |  |  | 10000 | Cx | 3 | 9 |
|  | Stream |  |  |  |  |  |  |  |  |  | |  |  |  |  |  |  |  |  |  |  |  |  |  |
|  |  | Grab |  |  |  |  |  |  |  |  | |  |  |  |  |  |  |  |  |  |  |  |  |  |
|  |  |  | MF |  |  |  |  |  |  |  | |  |  |  |  |  |  |  |  |  |  |  |  |  |
|  |  |  |  |  | 10000 | Cx | 148 | 348 |  | 1000 | | Cx | 18 | 29 |  | 1000 | Cx | 7 | 29 |  | 10000 | Cx | 25 | 29 |
|  |  |  |  |  |  |  |  |  |  | 9000 | | Cx | 3 | 29 |  | 9000 | Cx | 1 | 29 |  |  |  |  |  |
|  |  |  | MMS |  | 10000 | Cx | 11 | 29 |  | 10000 | | Cx | 142 | 348 |  | 10000 | Cx | 39 | 347 |  | 10000 | Cx | 233 | 348 |
|  |  | Moore Swab | - |  |  | Cx | 50 | 88 |  |  | | Cx | 23 | 86 |  |  | Cx | 6 | 86 |  |  | Cx | 77 | 88 |
| RICH | |  |  |  |  |  |  |  |  |  | |  |  |  |  |  |  |  |  |  |  |  |  |  |
|  | Lake or Reservoir ^h^ | Grab | MF |  | 10000 | Cx | 1 | 16 |  |  | |  |  |  |  |  |  |  |  |  | 10000 | Cx | 2 | 16 |
|  | Ocean | Grab | MF |  | 10000 | Cx | 0 | 6 |  |  | |  |  |  |  |  |  |  |  |  | 10000 | Cx | 0 | 6 |
|  | River | Grab | MF |  | 10000 | Cx | 3 | 30 |  |  | |  |  |  |  |  |  |  |  |  | 10000 | Cx | 28 | 30 |
|  | Stream | Grab | MF |  | 10000 | Cx | 0 | 13 |  |  | |  |  |  |  |  |  |  |  |  | 10000 | Cx | 7 | 13 |
| RUF | |  |  |  |  |  |  |  |  |  | |  |  |  |  |  |  |  |  |  |  |  |  |  |
|  | Canal | Grab | TFF |  | 10000 | Cx | 57 | 58 |  |  | |  |  |  |  |  |  |  |  |  |  |  |  |  |
|  | Lake or Reservoir | Grab | TFF |  | 10000 | Cx | 24 | 24 |  |  | |  |  |  |  |  |  |  |  |  |  |  |  |  |
|  | Pond | Grab | TFF |  | 10000 | Cx | 12 | 12 |  |  | |  |  |  |  |  |  |  |  |  |  |  |  |  |
|  | River | Grab | TFF |  | 10000 | Cx | 12 | 12 |  |  | |  |  |  |  |  |  |  |  |  |  |  |  |  |
|  | Stream | Grab | TFF |  | 10000 | Cx | 96 | 96 |  |  | |  |  |  |  |  |  |  |  |  |  |  |  |  |
| SFBR | |  |  |  |  |  |  |  |  |  | |  |  |  |  |  |  |  |  |  |  |  |  |  |
|  | River | Grab | MF |  | 200 | CIDT | 83 | 111 |  | 1000 | | CIDT | 88 | 126 |  |  |  |  |  |  | 200 | CIDT | 60 | 96 |
| SHV | |  |  |  |  |  |  |  |  |  | |  |  |  |  |  |  |  |  |  |  |  |  |  |
|  | River | Grab | MF |  | 5 | Cx | 6 | 15 |  |  | |  |  |  |  |  |  |  |  |  |  |  |  |  |
|  | Stream | Grab | MF |  | 5 | Cx | 1 | 2 |  |  | |  |  |  |  |  |  |  |  |  |  |  |  |  |
| SPW | |  |  |  |  |  |  |  |  |  | |  |  |  |  |  |  |  |  |  |  |  |  |  |
|  | Estuary or Tidal River | Grab | MF |  | 444 | Cx | 7 | 54 |  |  | |  |  |  |  |  |  |  |  |  | 90 | Cx | 7 | 54 |
|  | River | Grab | MF |  | 444 | Cx | 21 | 79 |  |  | |  |  |  |  |  |  |  |  |  | 90 | Cx | 10 | 79 |
|  | Stream | Grab | MF |  | 444 | Cx | 46 | 108 |  |  | |  |  |  |  |  |  |  |  |  | 90 | Cx | 20 | 108 |
| SRB | |  |  |  |  |  |  |  |  |  | |  |  |  |  |  |  |  |  |  |  |  |  |  |
|  | River | Grab | MMS |  | 10000 | Cx | 48 | 88 |  |  | |  |  |  |  |  |  |  |  |  |  |  |  |  |
|  | Stream | Grab | MMS |  | 10000 | Cx | 14 | 24 |  |  | |  |  |  |  |  |  |  |  |  |  |  |  |  |
| SUF | |  |  |  |  |  |  |  |  |  | |  |  |  |  |  |  |  |  |  |  |  |  |  |
|  | Canal | Grab | None |  | 33.3 | Cx | 27 | 96 |  |  | |  |  |  |  |  |  |  |  |  |  |  |  |  |
| TUF | |  |  |  |  |  |  |  |  |  | |  |  |  |  |  |  |  |  |  |  |  |  |  |
|  | Pond | Grab | None |  | 250 | Cx | 118 | 400 |  |  | |  |  |  |  |  |  |  |  |  |  |  |  |  |
| UBC | |  |  |  |  |  |  |  |  |  | |  |  |  |  |  |  |  |  |  |  |  |  |  |
|  | Canal | Grab | MF |  | 25 | Cx | 12 | 446 |  |  | |  |  |  |  | 25 | Cx | 46 | 446 |  | 25 | Cx | 22 | 446 |
| UNC | |  |  |  |  |  |  |  |  |  | |  |  |  |  |  |  |  |  |  |  |  |  |  |
|  | Pond | Grab | None |  | 333.3 | Cx | 33 | 45 |  |  | |  |  |  |  |  |  |  |  |  |  |  |  |  |
|  | Runoff | Grab | None |  | 333.3 | Cx | 9 | 9 |  |  | |  |  |  |  |  |  |  |  |  |  |  |  |  |
|  | Stream | Grab | None |  | 333.3 | Cx | 26 | 31 |  |  | |  |  |  |  |  |  |  |  |  |  |  |  |  |
| USGS | |  |  |  |  |  |  |  |  |  | |  |  |  |  |  |  |  |  |  |  |  |  |  |
|  | Ground-water | Grab | MF |  | 450 | Cx | 10 | 16 |  |  | |  |  |  |  |  |  |  |  |  |  |  |  |  |
|  | Stream | Grab | MF |  | 140 | Cx | 9 | 9 |  |  | |  |  |  |  |  |  |  |  |  |  |  |  |  |
| WIBCH | |  |  |  |  |  |  |  |  |  | |  |  |  |  |  |  |  |  |  |  |  |  |  |
|  | Lake or Reservoir |  |  |  |  |  |  |  |  |  | |  |  |  |  |  |  |  |  |  |  |  |  |  |
|  |  | Grab | None |  |  | Cx | 230 | 230 |  |  | |  |  |  |  |  |  |  |  |  |  |  |  |  |
|  |  | Grab | None |  |  | CIDT | 0 | 10 |  |  | |  |  |  |  |  |  |  |  |  |  |  |  |  |
| WIDNR | |  |  |  |  |  |  |  |  |  | |  |  |  |  |  |  |  |  |  |  |  |  |  |
|  | River |  |  |  |  |  |  |  |  |  | |  |  |  |  |  |  |  |  |  |  |  |  |  |
|  |  | Grab | None |  |  | Cx | 76 | 76 |  |  | |  |  |  |  |  |  |  |  |  |  |  |  |  |
|  |  | Grab | None |  |  | CIDT | 0 | 1 |  |  | |  |  |  |  |  |  |  |  |  |  |  |  |  |
|  | Stream |  |  |  |  |  |  |  |  |  | |  |  |  |  |  |  |  |  |  |  |  |  |  |
|  |  | Grab | None |  |  | Cx | 37 | 37 |  |  | |  |  |  |  |  |  |  |  |  |  |  |  |  |
|  |  | Grab | None |  |  | CIDT | 0 | 6 |  |  | |  |  |  |  |  |  |  |  |  |  |  |  |  |
| ZUF | |  |  |  |  |  |  |  |  |  | |  |  |  |  |  |  |  |  |  |  |  |  |  |
|  | Pond | Grab | MF |  | 150 | CIDT | 26 | 540 |  |  | |  |  |  |  |  |  |  |  |  | 150 | CIDT | 495 | 540 |

^a^ Filtration method used to filter grab samples: MF=membrane filtration; MMS= modified Moore swab; TTF= tangential flow filtration. Note Moore swabs are not filtered and are therefore marked with a “-“.

^b^ Volume in mL

^c^ Was a culture-based (Cx) or molecular (culture independent testing; CIDT) method used to detect the microbial target? MG indicates that a metagenomic method was used.

^d^ The number of samples that were positive (Pos.) for the microbial target and the total (Tot.) number samples that were tested for the microbial target.

^e^ Missing values indicates that samples were not tested for the given microbial target (e.g., ANF did not test for *Listeria* spp. or *L. monocytogenes*).

^f^ Samples collected by the FSL studies from reservoirs and lakes, including 12 samples tested for *Listeria* spp. and *L. monocytogenes* collected from the Great Lakes.

^g^ Samples collected by the FSL studies from ponds , including 9 samples tested for *Listeria* spp. and *L. monocytogenes* collected from urban ponds.

^h^ Samples collected by the RICH studies from reservoirs and lakes, including 10 samples tested for *Salmonella* and pathogenic *E. coli* collected from the Great Lakes.

**Supplemental Table 3:** Variance partitioning analysis results for foodborne pathogens

| Model Set | Factor Sets |  | Conditional Variance (Marginal Variance) ^a b^ | | | | | | | |
| --- | --- | --- | --- | --- | --- | --- | --- | --- | --- | --- |
|  |  |  |  |  |  |  |  |  |  |  |
|  |  |  | *Salmonella* |  | *Listeria* | |  | *E. coli* | | |
|  |  |  |  |  | *Listeria* spp. | *L. monocytogenes* | | Any Pathogenic *E. coli* | EPEC | STEC |
| One |  |  |  |  |  |  |  |  |  |  |
|  | Non-Methods ^c^ | |  |  | 0.13 (0.31) * | 0.18 (0.26) *** |  | 0.09 (0.64) *** | 0.15 (0.61) ** | 0.21 (0.37) *** |
|  | Methods ^d^ |  | < 0.01 (0.18) *** |  | 0.02 (0.01) *** | < 0.01 (0.08) |  | < 0.01 (0.55) | < 0.01 (0.47) *** | < 0.01 (0.16) *** |
|  | Residual Variance | |  |  | 0.69 (0.31) | 0.74 (0.26) |  | 0.36 (0.64) | 0.38 (0.62) | 0.63 (0.37) |
| Two |  |  |  |  |  |  |  |  |  |  |
|  | Region ^e^ |  | < 0.01 (0.13) |  | < 0.01 (0.09) | < 0.01 (0.13) |  | < 0.01 (0.48) | < 0.01 (0.58) | < 0.01 (0.26) |
|  | Temporal ^f^ |  | < 0.01 (< 0.01) *** |  | 0.01 (0.01) *** | 0.03 (0.06) *** |  | < 0.01 (0.01) | 0.02 (0.31) *** | < 0.01 (0.02) |
|  | Waterway (Set 1) ^g^ | |  |  | 0.04 (0.26) *** | 0.05 (0.21) *** |  | 0.02 (0.61) *** | 0.03 (0.62) *** | 0.06 (0.34) *** |
|  | Methods ^d^ |  | < 0.01 (0.18) *** |  | 0.03 (0.03) *** | 0.01 (0.07) *** |  | 0.02 (0.58) *** | < 0.01 (0.47) *** | 0.01 (0.16) *** |
|  | Residual Variance | |  |  | 0.73 (0.27) | 0.77 (0.23) |  | 0.37 (0.63) | 0.36 (0.64) | 0.65 (0.35) |
| Three |  |  |  |  |  |  |  |  |  |  |
|  | Waterway (Set 2) ^h^ | |  |  | 0.06 (0.26) *** | 0.10 (0.20) *** |  | 0.03 (0.61) *** | 0.04 (0.62) *** | 0.15 (0.34) *** |
|  | Temporal ^f^ |  | < 0.01 (< 0.01) *** |  | 0.01 (0.01) ** | 0.03 (0.06) *** |  | < 0.01 (0.01) | 0.02 (0.31) | < 0.01 (0.02) |
|  | Water Type ^i^ | |  |  | < 0.01 (0.10) | < 0.01 (0.05) |  | < 0.01 (0.15) | < 0.01 (0.40) *** | < 0.01 (0.03) |
|  | Methods ^d^ |  | 0.01 (0.18) *** |  | 0.03 (0.03) *** | 0.01 (0.07) *** |  | 0.02 (0.58) *** | < 0.01 (0.47) *** | 0.01 (0.16) *** |
|  | Residual Variance | |  |  | 0.73 (0.27) | 0.77 (0.23) |  | 0.37 (0.63) | 0.36 (0.64) | 0.65 (0.35) |

**^a^**  The number of stars indicates the *P-value* returned by redundancy analysis where the given matrix is the constraining variable and all other matrices are included as conditioning variables. Specifically, *** indicates *P* < 0.01, ** indicates *P* <0.01, and * indicates *P* <0.05.

^b^ Conditional variance represents the variance uniquely attributable to the given matrix, while marginal variance represents total variance or variance that is uniquely attributable to the matrix plus variance jointly attributable to the matrix and at least one other matrix.

^c^ Includes all non-methodological factors, including waterway, sampling site, water type (fine, mid, coarse and general), freshwater status, state, year, and season.

^d^ Includes all methodological factors, such as sample type, filter type, detection methods (culture versus molecular), and if a given gene was used for confirmation or detection.

^e^ Matrix representing state and other “region” factors, such as USDA region and Census Region.

^f^ Matrix representing temporal factors, namely year and season.

^g^ Matrix representing waterway that includes water type (fine, mid, coarse and general), freshwater status, waterway, and sampling site.

^h^ Matrix representing waterway that includes waterway and sampling site but not water type (fine, mid, coarse and general) and freshwater status.

^i^ Matrix representing water type that includes water type (fine, mid, coarse and general), and freshwater status. See footnote *h* in Supplemental Table 9 for a description of the four, nested water type classification schemes used here.

**Supplemental Table 4:** Variable importance rankings for factors included in foodborne pathogen conditional forests that were built using methodological, spatial, and temporal features to understand the relative impact of methodological differences (as opposed to spatial and temporal factors) on observed water quality. Prior to training the forest, separate generalized linear random effects model were fit to model likelihood of detecting each pathogen or pathogen surrogate using random effects of site nested in waterway; the dependent variable in each conditional forest was the residuals from these regression models.

|  | | Conditional Variable Importance | | | | | | | | |
| --- | --- | --- | --- | --- | --- | --- | --- | --- | --- | --- |
|  |  | *Salmonella* |  | *Listeria* | |  | Pathogenic *E. coli* ^a^ | | | |
|  |  |  |  | *Listeria* spp. | *L. monocytogenes* |  | pEC | EPEC | STEC | O157 |
| Detection Method ^b^ | | 1.54*10^-4^ |  | - | 6.92*10^-6^ |  | 2.54*10^-3^ | 2.54*10^-4^ | 3.14*10^-3^ | 3.79*10^-6^ |
| Filter Type ^c^ | | 5.51*10^-3^ |  | 1.14*10^-3^ | 4.39*10^-4^ |  | 2.20*10^-3^ | 3.53*10^-3^ | 1.19*10^-3^ | - |
| Freshwater Status ^d^ | | -1.92*10^-6^ |  | -1.14*10^-6^ | 0.00 |  | -6.82*10^-6^ | 0.00 | -8.93*10^-7^ | 2.01*10^-10^ |
| Gene Target ^e^ | | 6.90*10^-4^ |  | 1.21*10^-4^ | 9.31*10^-5^ |  | 9.78*10^-4^ | - | 9.78*10^-4^ | 5.81*10^-6^ |
| Sample Type ^f^ | | 7.72*10^-4^ |  | 1.93*10^-3^ | 1.22*10^-3^ |  | 2.60*10^-4^ | 2.21*10^-3^ | 6.73*10^-4^ | - |
| Sample Volume ^g^ | | 3.19*10^-3^ |  | 5.71*10^-4^ | 9.50*10^-4^ |  | 6.77*10^-4^ | 2.89*10^-3^ | 1.33*10^-3^ | 4.95*10^-6^ |
| Season | | 4.65*10^-3^ |  | 2.81*10^-3^ | 2.33*10^-2^ |  | 7.53*10^-4^ | 2.14*10^-2^ | 1.58*10^-3^ | 2.58*10^-4^ |
| State | | 2.75*10^-3^ |  | 2.38*10^-3^ | 1.78*10^-3^ |  | 7.73*10^-3^ | 1.06*10^-2^ | 5.55*10^-3^ | 9.88*10^-6^ |
| Water Type ^h^ | |  |  |  |  |  |  |  |  |  |
|  | Fine-Scale | 4.00*10^-4^ |  | 3.13*10^-4^ | 1.10*10^-3^ |  | 2.01*10^-4^ | 2.89*10^-3^ | 4.84*10^-5^ | 2.57*10^-5^ |
|  | Mid-Scale | 3.57*10^-4^ |  | 4.66*10^-4^ | 8.37*10^-4^ |  | 1.59*10^-4^ | 1.67*10^-3^ | 6.31*10^-5^ | 1.59*10^-5^ |
|  | Coarse-Scale | -1.20*10^-6^ |  | 5.84*10^-4^ | 0.00 |  | -8.11*10^-6^ | -2.15*10^-6^ | 1.34*10^-6^ | 2.59*10^-11^ |
|  | General | 5.96*10^-4^ |  | - | 5.14*10^-5^ |  | 1.42*10^-4^ | 4.81*10^-3^ | 1.86*10^-4^ | 2.35*10^-6^ |
| Year | | 2.73*10^-3^ |  | 1.13*10^-3^ | 8.49*10^-4^ |  | 3.05*10^-4^ | 2.98*10^-3^ | 7.84*10^-4^ | 4.57*10^-4^ |

**^a^** pEC indicates any samples positive for any type of pathogenic *E. coli*. EPEC indicates samples positive for enteropathogenic *E. coli* (based on detection of the *eaeA*). O157 indicates samples positive for *E. coli* O157. STEC indicates samples positive for Shiga-toxin producing *E. coli* (based on detection if *stx*).

^b^ Was target detection performed using a molecular or culture-based method.

^c^ Type of filter used to process grab samples (membrane filter, modified Moore swab, tangential flow filtration and/or no filtration); if a Moore swab was collected than this factor was set equal to Moore swab.

^d^ If the water sample represents fresh water or brackish/saltwater.

^e^ The gene target used for molecular confirmation following culture-based detection, PCR-screen prior to culture-based detection, or for molecular detection when molecular (as opposed to culture-based detection) was performed. For *Listeria* spp. and *L. monocytogenes* this factor indicates if *sigB* was used for culture confirmation or not. Some studies confirmed detection of *L. monocytogenes* using biochemical assays, the variable importance (VI) measure for this factor was 1.45*10^-4^. Five different gene targets were used for detection and/or confirmation of pathogenic *E. coli*. EPEC and STEC detection were based on detection of *eaeA* and *stx*, respectively, and therefore this factor was constant and not included as a feature in the EPEC and STEC forest. The value in the table for pEC and O157 is the VI for if *stx* was used a gene target. Additionally, pEC detection was performed using *eaeA* (VI=7.32*10^-3^), *flic* (VI=4.10*10^-4^), *hly* (VI=1.03*10^-3^), and *rfb* (VI=1.03*10^-3^), while O157 detection was performed using *eaeA* (VI=1.24*10^-4^), *flic* (VI=8.22*10^-4^), and *hly* (VI=1.05*10^-4^). The gene target used for *Salmonella* detection was either *invA* (reported in table) or *ttr* (VI=0.00).

^f^ Was the sample collected a grab sample or a Moore swab.

^g^ The volume of the grab sample; if a Moore swab was collected volume was set to 10,000L.

^h^ Four levels of water type were considered in this study. The finest scale variable (fine-scale water type) included 27 levels: canals, channelized streams, ditches, enclosed stormwater infrastructure (e.g., storm drains), ephemeral streams, estuaries and tidal rivers, facility or municipal water, floodwater, free-standing water tanks, the Great Lakes, groundwater, lakes or reservoirs, leachate and mining discharge, manure or wastewater, non-tidal rivers, non-tidal streams, ocean, ponds, rain, reclaimed or treated wastewater, run-off, salt ponds, seeps, springs, tidal streams, urban ponds fed by municipal water, and wetlands. Mid-scale water type included 20 levels : canals, estuaries and tidal rivers, facility or municipal water, the Great Lakes, groundwater, lakes or reservoirs, leachate and mining discharge, manure or wastewater, non-tidal rivers, non-tidal streams (including channelized and ephemeral streams), ocean, other water types (e.g., freestanding water tanks, rain), ponds (including urban ponds), reclaimed or treated wastewater, run-off (including floodwaters), salt ponds, seeps, stormwater infrastructure (including ditches, enclosed stormwater infrastructure), tidal streams, and wetlands. Coarse-scale water type included 10 levels : canals, facility influent or effluent (e.g., municipal water and treated/reclaimed wastewater), groundwater (including seeps, leachate and mining discharge), manure or wastewater, estuaries/oceans/tidal rivers, other water types (e.g., free-standing water tanks, rain), lakes/ponds/reservoirs (including salt ponds and the Great Lakes), run-off (including floodwater, ditches and enclosed stormwater infrastructure), streams and rivers (including channelized streams, ephemeral streams, springs, and tidal streams), and wetlands. General-scale water type included four levels : groundwater, manure or wastewater, surface water (including canals, estuaries, lakes, oceans, ponds, reservoirs, rivers, run-off, streams, and wetlands). The water type variables are nested. For example, urban ponds (fine-scale water type) collapse into ponds (mid-scale), which collapse into lakes/ponds/reservoirs (coarse-scale), which collapses into surface water (general).

**Supplemental Table 5:** Results of generalized linear mixed models that were implemented to quantify how using a given methodological approach affected likelihood of pathogen or pathogen surrogate detection^a^

|  |  | Comparison | Reference ^b^ | Odds ^c^ | SD ^d^ | *P*-value ^e^ |
| --- | --- | --- | --- | --- | --- | --- |
| *Salmonella* | |  |  |  |  |  |
|  | Confirmation Method ^g^ | *invA* | Not *invA* | 0.16 | 1.19 | < 0.001 |
|  |  | *ttr* | Not *ttr* | 0.05 | 1.74 | < 0.001 |
|  | Detection Method ^k^ | Molecular | Culture | 0.02 | 1.30 | < 0.001 |
|  | Sample Filter ^h^ | |  |  |  |  |
|  |  | mMS | MF | 4.70 | 1.26 | < 0.001 |
|  |  | MS | MF | 6.25 | 1.27 | < 0.001 |
|  |  | No Filter | MF | 2.73 | 1.32 | < 0.001 |
|  |  | TFF | MF | 3394.00 | 2.99 | < 0.001 |
|  |  | MS | mMS | 1.33 | 1.25 | 0.198 |
|  |  | No Filter | mMS | 0.58 | 1.43 | 0.147 |
|  |  | TFF | mMS | 723.00 | 3.06 | < 0.001 |
|  |  | No Filter | MS | 0.44 | 1.44 | 0.031 |
|  |  | TFF | MS | 543.00 | 3.07 | < 0.001 |
|  |  | TFF | No Filter | 1241.00 | 2.96 | < 0.001 |
|  | Sample Type ^i^ | MS | GS | 2.19 | 1.27 | 0.001 |
|  | Volume ^j^ |  | | 1.64 | 1.45 | < 0.001 |
| *Listeria* spp. | |  |  |  |  |  |
|  | Gene Target ^g^ | *sigB* | Not *sigB* | 0.06 | 4.24 | 0.057 |
|  | Sample Filter ^h^ |  |  |  |  |  |
|  |  | mMS | MF | 1.07 | 1.30 | 0.801 |
|  |  | MS | MF | 0.37 | 1.46 | 0.016 |
|  |  | MS | mMS | 0.35 | 1.36 | 0.002 |
|  | Sample Type i^i^ | MS | GS | 0.35 | 1.35 | 0.001 |
|  | Volume ^j^ |  |  | 0.77 | 0.08 | 0.002 |
| *L. monocytogenes* | |  |  |  |  |  |
|  | Confirmation Method ^g^ |  |  |  |  |  |
|  |  | *hlyA* | BC | 3.94 | 6.06 | 0.669 |
|  |  | *sigB* | BC | 1.75 | 5.61 | 0.744 |
|  |  | *sigB* | *hlyA* | 0.45 | 1.72 | 0.402 |
|  | Gene Target ^g^ | *sigB* | Not *sigB* | 0.50 | 1.68 | 0.177 |
|  | Detection Method ^k^ | Molecular | Culture | 2.25 | 1.72 | 0.134 |
|  | Sample Filter ^h^ |  |  |  |  |  |
|  |  | mMS | MF | 0.97 | 1.35 | 0.924 |
|  |  | MS | MF | 0.65 | 1.65 | 0.458 |
|  |  | No Filter | MF | 7.58 | 2.37 | 0.048 |
|  |  | MS | mMS | 0.67 | 1.54 | 0.458 |
|  |  | No Filter | mMS | 7.80 | 2.49 | 0.048 |
|  |  | No Filter | MS | 11.70 | 2.71 | 0.048 |
|  | Sample Type ^i^ | MS | GS | 0.67 | 1.54 | 0.344 |
|  | Volume ^j^ |  |  | 0.92 | 0.11 | 0.047 |
| Pathogenic *E. coli ^l^* | |  |  |  |  |  |
|  | Confirmation Method ^g^ | | | | | |
|  |  |  |  |  |  |  |
|  |  | *eaeA* | Not *eaeA* | 752.00 | 1.68 | <0.001 |
|  |  | *flic* | Not *flic* | 12.50 | 4.63 | 0.212 |
|  |  | *hly* | Not *hly* | 72.60 | 1.84 | <0.001 |
|  |  | *rfb* | Not *rfb* | 0.09 | 1.48 | <0.001 |
|  |  | *stx* | Not *stx* | 983.00 | 1.76 | <0.001 |
|  | Detection Method ^k^ | Molecular | Culture | 52.40 | 1.39 | < 0.001 |
|  | Sample Filter ^h^ | |  |  |  |  |
|  |  | mMS | MF | 51.20 | 1.49 | < 0.001 |
|  |  | MS | MF | 8.01 | 1.57 | < 0.001 |
|  |  | No Filter | MF | <0.01 | 8.68 | 0.012 |
|  |  | MS | mMS | 0.16 | 1.58 | < 0.001 |
|  |  | No Filter | mMS | <0.01 | 8.99 | < 0.001 |
|  |  | No Filter | MS | <0.01 | 9.16 | < 0.001 |
|  | Sample Type ^i^ | MS | GS | 1.05 | 1.55 | 0.908 |
|  | Volume ^j^ |  | | 1.80 | 0.13 | < 0.001 |
| Enteropathogenic *E. coli* | | |  |  |  |  |
|  | Detection Method ^k^ | Molecular | Culture | 6.23 | 1.49 | < 0.001 |
|  | Sample Filter ^h^ | |  |  |  |  |
|  |  | mMS | MF | 7.10 | 1.48 | < 0.001 |
|  |  | MS | MF | 10.30 | 1.97 | 0.001 |
|  |  | No Filter | MF | < 0.01 | 5.60 | < 0.001 |
|  |  | MS | mMS | 1.45 | 1.86 | 0.550 |
|  |  | No Filter | mMS | < 0.01 | 5.75 | < 0.001 |
|  |  | No Filter | MS | < 0.01 | 6.27 | < 0.001 |
|  | Sample Type ^i^ | MS | GS | 3.19 | 1.83 | 0.055 |
|  | Volume ^j^ |  |  | 1.25 | 0.20 | 0.265 |
| *E. coli* O157:H7 ^k^ | |  |  |  |  |  |
|  | Volume ^j^ |  |  | 0.31 | 6.94 | 0.866 |
| Shiga-toxin producing *E. coli* | |  |  |  |  |  |
|  | Detection Method ^k^ | Molecular | Culture | 3.90 | 1.24 | < 0.001 |
|  | Sample Filter ^h^ |  |  |  |  |  |
|  |  | mMS | MF | 1.18 | 1.35 | 0.059 |
|  |  | MS | MF | 1.57 | 1.42 | 0.029 |
|  |  | No Filter | MF | 0.00 | 2.35 | < 0.001 |
|  |  | MS | mMS | 1.34 | 1.34 | 0.039 |
|  |  | No Filter | mMS | 0.00 | 2.32 | < 0.001 |
|  |  | No Filter | MS | 0.00 | 2.43 | < 0.001 |
|  | Sample Type ^i^ | MS | GS | 2.20 | 1.31 | 0.004 |
|  | Volume ^j^ |  |  | 1.18 | 0.07 | 0.019 |

**^a^** The models were implemented with the binomial family, a logit link, random effects of site nested in waterway nested in state, a random effect of season, and a fixed effect for the methodological variable of interest. To determine which levels of each methodological variable were and were not associated with significant differences in the likelihood of detecting the target pathogen or surrogate, Tukey’s HSD was performed using the multcomp package. These analyses were only performed with likelihood of pathogen and pathogen surrogates detection as the outcome (as opposed to likelihood of host-specific fecal source tracking marker detection, or log10 FIB concentration) since (i) pathogen contamination is the primary outcome of practical interest (e.g., fecal indicators are used in monitoring and risk mitigation to identify when a water source may have been fecally contaminated and thus more likely to be contaminated by foodborne pathogens), and (ii) methodological variables were consistently among the top-ranked factors in the pathogen and pathogen surrogate conditional forests but not in the fecal indicator bacteria forests.

^b^ The reference-level for categorical factors. The effect estimate (EE) should be interpreted as, “the change in odds of target detection for associated with shifting from using the reference methods to the methods listed under “comparison”. Volume is continuous so does not have reference or comparison categories.

^c^ Effect estimate

^d^ Standard deviation

^e^ P-value after implementing the Benjamin-Hochberg correction for multiple comparison correction.

^f^ For grab samples (GS) was the grab sample filtered or not, and it was filtered was it filtered through a membrane filter (MF), through a modified Moore swabs (mMS), or using tangential flow filtration (TFF). If the sample collected was a Moore swab (MS) then filter type was set equal to MS.

^g^  The gene target used for molecular confirmation following culture-based detection, PCR-screen prior to culture-based detection, or for molecular detection when molecular (as opposed to culture-based detection) was performed. A subset of *L. monocytogenes* used biochemical analyses (BC) as opposed to PCR-based screens for specific genes to confirm presumptive *L. monocytogenes* positive samples as positive. See Supplemental Table 7 footnote *e*.

^h^ Type of filter used to process grab samples (membrane filter, modified Moore swab, tangential flow filtration and/or no filtration); if a Moore swab was collected than this factor was set equal to Moore swab.

^i^ Was the sample collected a grab sample or a Moore swab.

^j^ The volume of the grab sample; if a Moore swab was collected volume was set to 10,000 L.

^k^ Was target detection performed using a molecular or culture-based method. All samples where culture-based methods were used for *E. coli* O157 detection were *E. coli* O157-negative (N=215) but 5% (N=61) of samples where molecular methods were used for *E. coli* O157 detection were *E. coli* O157-positive. As a result, a model for *E. coli* O157 detection method could not be run. However, if 1 sample where culture-based detection was used had been *E. coli* O157-positive then the odds of *E. coli* O157 detection would have been approx. 12 [(61/1093)/(1/214)] times greater using molecular compared to culture-based methods.

**Supplemental Table 6:** Variable importance rankings for factors included in conditional forests that were built to determine if region ^a^ or water type ^b^ were associated more strongly with each microbial target after accounting for other confounding factors, and which regional scheme was most strongly associated with likelihood of detecting foodborne pathogens or pathogen surrogates. Prior to training the forest, separate generalized linear random effects models were fit to model the likelihood of detecting each pathogen using random effects for each methodological factor available for the given target; the dependent variable in the conditional forests was the residuals from these regression models.

|  |  | Conditional Variable Importance | | | | | | | | |
| --- | --- | --- | --- | --- | --- | --- | --- | --- | --- | --- |
|  |  | *Salmonella* |  | *Listeria spp.* | |  | Pathogenic *E. coli* | | | |
|  |  |  |  | *Listeria* spp. | *L. monocytogenes* |  | Pathogenic *E. coli* | EPEC | STEC | O157 |
| Agricultural Region | |  |  |  |  |  |  |  |  |  |
|  | Scheme 1 | 1.21*10^-4^ |  | 8.77*10^-4^ | 1.00*10^-3^ |  | 1.88*10^-4^ | 8.39*10^-4^ | 1.19*10^-3^ | 1.57*10^-4^ |
|  | Scheme 2 | 5.75*10^-5^ |  | 1.33*10^-3^ | 6.07*10^-4^ |  | 2.69*10^-4^ | 8.33*10^-4^ | 4.75*10^-4^ | 6.57*10^-5^ |
|  | Scheme 3 | 4.89*10^-4^ |  | 2.05*10^-3^ | 2.59*10^-3^ |  | 3.63*10^-3^ | 2.88*10^-4^ | 1.39*10^-3^ | 3.27*10^-5^ |
|  | Scheme 4 | 4.11*10^-5^ |  | 8.86*10^-4^ | 5.63*10^-4^ |  | 3.99*10^-4^ | 3.36*10^-4^ | 3.04*10^-4^ | 5.55*10^-5^ |
| Biome | | -1.44*10^-6^ |  |  |  |  |  |  |  |  |
| Census Region | | 1.82*10^-4^ |  | 3.07*10^-3^ | 1.68*10^-3^ |  | 5.21*10^-4^ | 9.66*10^-4^ | 3.26*10^-4^ | 7.99*10^-5^ |
| Climate Region | | 9.01*10^-4^ |  | 8.78*10^-4^ | 8.07*10^-4^ |  | 2.07*10^-4^ | 6.91*10^-4^ | 7.73*10^-4^ | 1.16*10^-4^ |
| Ecoregion | |  |  |  |  |  |  |  |  |  |
|  | Level I | 1.06*10^-3^ |  | 9.55*10^-4^ | 2.76*10^-4^ |  | 2.58*10^-4^ | 1.56*10^-4^ | 4.17*10^-4^ | 1.26*10^-5^ |
|  | Level II | 2.58*10^-3^ |  | 6.35*10^-4^ | 3.94*10^-4^ |  | 1.73*10^-3^ | 2.07*10^-3^ | 7.83*10^-4^ | 9.77*10^-5^ |
| EPA Region | | 2.23*10^-4^ |  | 2.68*10^-3^ | 2.79*10^-3^ |  | 8.55*10^-4^ | 2.07*10^-3^ | 1.83*10^-3^ | 6.85*10^-5^ |
| Freshwater Status | | 6.94*10^-5^ |  | 3.82*10^-5^ | 0.00 |  | 0.00 | 0.00 | 0.00 | 0.00 |
| Habitat Type | |  |  |  |  |  |  |  |  |  |
|  | Aquatic | 3.22*10^-4^ |  | 2.50*10^-4^ | 5.58*10^-4^ |  | 7.92*10^-5^ | 2.63*10^-3^ | 1.95*10^-4^ | 2.22*10^-4^ |
|  | Terrestrial | 8.74*10^-5^ |  | 5.87*10^-4^ | 1.87*10^-5^ |  | 5.23*10^-4^ | 1.24*10^-3^ | 1.33*10^-4^ | 1.40*10^-4^ |
| Hydrological Region | | 3.98*10^-4^ |  | 2.01*10^-3^ | 1.22*10^-3^ |  | 1.02*10^-3^ | 2.73*10^-4^ | 1.87*10^-4^ | 4.67*10^-5^ |
| State | | 1.04*10^-3^ |  | 3.29*10^-3^ | 3.03*10^-3^ |  | 4.80*10^-3^ | 5.30*10^-4^ | 2.29*10^-3^ | 3.94*10^-5^ |
| USDA Region | | 5.25*10^-4^ |  | 1.72*10^-3^ | 1.51*10^-3^ |  | 9.20*10^-4^ | 2.12*10^-3^ | 1.37*10^-3^ | 1.75*10^-4^ |
| Water Type | |  |  |  |  |  |  |  |  |  |
|  | Fine-scale | 4.30*10^-3^ |  | 8.89*10^-3^ | 1.97*10^-2^ |  | 3.70*10^-3^ | 6.93*10^-3^ | 3.01*10^-3^ | 9.18*10^-6^ |
|  | Mid-scale | 4.13*10^-3^ |  | 1.04*10^-2^ | 1.83*10^-2^ |  | 3.43*10^-3^ | 6.42*10^-3^ | 2.14*10^-3^ | 9.01*10^-6^ |
|  | Coarse-scale | 6.85*10^-6^ |  | 8.12*10^-3^ | 7.35*10^-4^ |  | 8.01*10^-5^ | 9.61*10^-5^ | 5.04*10^-4^ | 1.68*10^-6^ |
|  | General | 1.94*10^-3^ |  | - ^c^ | -^c^ |  | 8.95*10^-4^ | 7.95*10^-3^ | 1.27*10^-3^ | 2.92*10^-6^ |

^a^ Using GPS coordinates and county, samples were separately classified into regions using 14 different regional schemes, including schemes based on biome, two nested ecoregion schemes, aquatic and terrestrial habitat type, interstate climate region, hydrologic region (based on USGS HUC2 unit codes), three regional schemes used by US federal agencies, and four schemes based on agricultural practices and/or output. For US-specific regional schemes, sites outside the US were assigned to the region of the closest US site.

^b^ Freshwater status as well as four levels of water type were considered in this study. See footnote *h* in Supplemental Table 9 for a description of the four, nested water type classification schemes used here.

^c^  The – indicates that the values for the given feature were constant for all samples with data for the given target. As such, that feature was not included in the feature set for forest development

**Supplemental Table 7:** Variable importance rankings for factors included in conditional forests that were built using methodological, spatial, and temporal features to understand the relative impact of methodological differences (as opposed to spatial and temporal factors) on observed water quality. Prior to training the forest, separate general linear random effects models were fit to model log10 concentration of each fecal indicator bacteria in water samples using random effects of site nested in waterway; the dependent variable in the conditional forests was the residuals from these regression models.

|  | | Conditional Variable Importance | | | | | | | | | | | |
| --- | --- | --- | --- | --- | --- | --- | --- | --- | --- | --- | --- | --- | --- |
|  |  | *E. coli* ^a b^ | | | | | *Enterococcus* ^c^ | Fecal Coliforms ^a d^ | | | | | Total Coliforms ^e^ |
|  |  | Canals | Lakes and Similar | River | Other | Stream |  | Canals | Lakes and Similar | River | Other | Stream |  |
| Freshwater Status ^f^ | | 9.48*10^-6^ | 0.00 | 0.00 | 6.78*10^-5^ | 0.00 | 3.49*10^-6^ | 3.96*10^-3^ | 1.38*10^-6^ |  | 6.73*10^-7^ | 0.00 | 2.27*10^-6^ |
| Media | | 5.31*10^-4^ | 4.82*10^-5^ | 2.28*10^-5^ | 2.83*10^-4^ | 2.70*10^-5^ | 9.46*10^-6^ | 2.19*10^-4^ | 1.48*10^-4^ | 3.65*10^-4^ | 2.60*10^-5^ | 8.35*10^-5^ | 7.63*10^-6^ |
| Method | |  |  |  |  |  |  |  |  |  |  |  |  |
|  | Fine-scale | 7.88*10^-4^ | 1.09*10^-4^ | 9.78*10^-5^ | 3.66*10^-4^ | 5.76*10^-5^ | 2.38*10^-4^ | 7.15*10^-4^ | 2.24*10^-4^ | 9.43*10^-5^ | 1.30*10^-3^ | 1.67*10^-4^ | 1.43*10^-5^ |
|  | Mid-scale | 7.93*10^-5^ | 4.16*10^-6^ | 1.26*10^-6^ | 2.08*10^-5^ | 7.55*10^-6^ | 1.17*10^-5^ | 2.00*10^-4^ | 2.51*10^-5^ | 1.05*10^-5^ | 5.26*10^-7^ | 6.29*10-6 | 8.62*10^-8^ |
|  | Coarse-scale | 2.05*10^-4^ | 2.66*10^-5^ | -1.97*10^-7^ | 2.61*10^-5^ | 1.36*10^-5^ | 3.01*10^-6^ | 1.79*10^-4^ | 2.85*10^-5^ | 2.62*10^-6^ | 2.60*10^-6^ | 2.28*10^-6^ | 2.31*10^-6^ |
| Season | | 3.90*10^-2^ | 3.24*10^-3^ | 3.15*10^-2^ | 6.52*10^-3^ | 3.30*10^-2^ | 2.28*10^-2^ | 2.94*10^-2^ | 1.03*10^-2^ | 2.86*10^-2^ | 1.19*10^-2^ | 2.89*10^-2^ | 2.51*10^-2^ |
| State | | 5.75*10^-3^ | 3.40*10^-3^ | 1.27*10^-2^ | 2.10*10^-3^ | 1.05*10^-2^ | 5.96*10^-3^ | 5.15*10^-3^ | 2.36*10^-3^ | 1.37*10^-2^ | 7.96*10^-4^ | 7.10*10^-3^ | 2.41*10^-2^ |
| Water Type ^g^ | |  |  |  |  |  |  |  |  |  |  |  |  |
|  | Fine-scale | - | 1.83*10^-5^ | - | 1.21*10^-3^ | 2.43*10^-6^ | 2.62*10^-4^ | - | 2.17*10^-4^ | - | 2.01*10^-5^ | 1.81*10^-5^ | 7.33*10^-5^ |
|  | Mid-scale | - | 1.02*10^-5^ | - | 7.03*10^-4^ | - | 1.21*10^-4^ | - | 1.04*10^-4^ | - | 1.37*10^-5^ | - | 3.97*10^-5^ |
|  | Coarse-scale | - | - | - | 3.05*10^-4^ | - | 0.00 | - | - | - | 4.47*10^-6^ | - | 1.69*10^-7^ |
|  | General | - | - | - | 8.06*10^-4^ | - | 1.12*10^-5^ | - | - | - | 4.98*10^-6^ | - | 4.88*10^-5^ |
| Year | | 3.15*10^-3^ | 2.31*10^-3^ | 1.80*10^-3^ | 1.03*10^-3^ | 1.45*10^-3^ | 2.07*10^-3^ | 2.22*10^-2^ | 7.76*10^-4^ | 5.46*10^-3^ | 7.70*10^-3^ | 1.53*10^-3^ | 2.63*10^-2^ |

^a^ Due to the number of samples with *E. coli* (N=) and fecal coliform data (N=), forests could not be run using all samples due to computational constraints. Instead, five, separate water type-specific models were implemented where *E. coli* and fecal coliform levels were the outcome.

^b^ FIB methods were categorized using three nested schemes. separate study-specific assignations were used if the method did not fall into any of these established methods. These fine-scales methods were then grouped in mid-scale methods. These mid-scale methods were then grouped in coarse-scale methods.

**^c^** FIB methods were categorized using three nested schemes. For *Enterococcus* separate study-specific assignations were used if the method did not fall into any of these established methods. These fine-scales methods were then grouped in mid-scale methods. These mid-scale methods were then grouped in coarse-scale methods.

**^d^** FIB methods were categorized using three nested schemes. For fecal coliforms separate study-specific assignations were used if the method did not fall into any of these established methods. These fine-scales methods were then grouped in mid-scale methods. These mid-scale methods were then grouped in coarse-scale methods.

**^e^** FIB methods were categorized using three nested schemes. For total coliforms separate study-specific assignations were used if the method did not fall into any of these established methods. These fine-scales methods were then grouped in mid-scale methods. These mid-scale methods were then grouped in coarse-scale methods.

**^f^** If the water sample represents fresh water or brackish/saltwater.

^g^ Four levels of water type were considered in this study. See footnote *h* in Supplemental Table 9 for a description of the four, nested water type classification schemes used here.

**Supplemental Table 8:** Variable importance rankings for factors included in conditional forests that were built to determine if region ^a^ or water type ^b^ were associated more strongly with the log10 concentration of each fecal indicator bacteria after accounting for other confounding factors, and which regional scheme was most strongly associated with each microbial target. Prior to training the forest, separate generalized linear random effects models were fit to model the log10 concentration of each fecal indicator bacteria in water samples using random effects for each methodological factor available for the given target; the dependent variable in the conditional forests was the residuals from these regression models.

|  | | Conditional Variable Importance | | | | | | | | | | | |
| --- | --- | --- | --- | --- | --- | --- | --- | --- | --- | --- | --- | --- | --- |
|  |  | *E. coli* | | | | | *Enterococcus* | Fecal Coliforms | | | | | Total Coliforms |
|  |  | Canals | Lakes and Similar | River | Other | Stream |  | Canals | Lakes and Similar | River | Other | Stream |  |
| Agricultural Region | |  |  |  |  |  |  |  |  |  |  |  |  |
|  | Scheme 1 | 8.16*10^-4^ | 2.98*10^-5^ | 1.55*10^-4^ | 6.55*10^-5^ | 4.09*10^-5^ | 1.59*10^-7^ | 5.11*10^-4^ | 1.17*10^-4^ | 4.01*10^-6^ | 1.07*10^-5^ | 5.61*10^-5^ | 1.24*10^-5^ |
|  | Scheme 2 | 4.26*10^-4^ | 8.16*10^-5^ | 7.53*10^-5^ | 2.00*10^-5^ | 1.03*10^-4^ | 7.46*10^-7^ | 3.09*10^-4^ | 1.80*10^-4^ | 3.59*10^-6^ | 3.13*10^-7^ | 5.98*10^-5^ | 6.75*10^-7^ |
|  | Scheme 3 | 1.77*10^-3^ | 4.09*10^-4^ | 1.31*10^-3^ | 2.09*10^-4^ | 3.53*10^-4^ | 3.52*10^-4^ | 9.50*10^-4^ | 3.64*10^-4^ | 2.57*10^-4^ | 1.13*10^-4^ | 5.69*10^-4^ | 2.97*10^-4^ |
|  | Scheme 4 | 1.94*10^-4^ | 6.28*10^-5^ | 1.12*10^-4^ | 4.45*10^-5^ | 7.85*10^-5^ | 7.51*10^-7^ | 1.18*10^-4^ | 8.98*10^-5^ | 4.41*10^-6^ | 9.74*10^-7^ | 1.06*10^-4^ | 0.00 |
| Biome | | 1.17*10^-4^ | 0.00 | 0.00 | 0.00 | 0.00 | 0.00 | 2.46*10^-5^ | 0.00 | 0.00 | 0.00 | 0.00 | 0.00 |
| Census Region | | 4.55*10^-4^ | 2.78*10^-4^ | 4.49*10^-4^ | 1.25*10^-5^ | 2.83*10^-4^ | 1.00*10^-5^ | 4.13*10^-4^ | 1.22*10^-4^ | 2.44*10^-5^ | 4.30*10^-5^ | 1.86*10^-4^ | 1.62*10^-5^ |
| Climate Region | | 1.31*10^-4^ | 1.49*10^-5^ | 1.41*10^-4^ | 3.21*10^-5^ | 2.69*10^-5^ | 5.95*10^-7^ | 2.98*10^-4^ | 3.37*10^-5^ | 7.96*10^-5^ | 3.64*10^-5^ | 5.24*10^-5^ | 1.43*10^-5^ |
| Ecoregion | |  |  |  |  |  |  |  |  |  |  |  |  |
|  | Level I | 4.57*10^-4^ | 1.43*10^-3^ | 3.24*10^-3^ | 5.12*10^-4^ | 1.08*10^-3^ | 6.68*10^-5^ | 1.71*10^-3^ | 9.20*10^-5^ | 3.00*10^-4^ | 7.64*10^-4^ | 7.00*10^-4^ | 1.04*10^-4^ |
|  | Level II | 1.11*10^-3^ | 5.45*10^-3^ | 5.21*10^-3^ | 2.20*10^-3^ | 6.12*10^-3^ | 5.28*10^-3^ | 3.45*10^-3^ | 7.94*10^-4^ | 6.23*10^-3^ | 5.26*10^-4^ | 3.29*10^-3^ | 2.63*10^-3^ |
| EPA Region | | 9.02*10^-4^ | 4.43*10^-4^ | 4.84*10^-4^ | 1.81*10^-4^ | 4.49*10^-4^ | 6.99*10^-6^ | 2.79*10^-4^ | 4.81*10^-4^ | 1.59*10^-4^ | 1.30*10^-5^ | 3.20*10^-4^ | 2.34*10^-5^ |
| Freshwater Status | | 0.00 | 0.00 | 0.00 | 1.39*10^-5^ | 0.00 | 1.67*10^-3^ | 1.52*10^-2^ | 2.39*10^-5^ |  | 1.12*10^-5^ | 0.00 | -3.25*10^-6^ |
| Habitat Type | |  |  |  |  |  |  |  |  |  |  |  |  |
|  | Aquatic | 1.75*10^-3^ | 8.38*10^-4^ | 5.04*10^-4^ | 4.38*10^-4^ | 8.77*10^-4^ | 5.15*10^-4^ | 2.88*10^-3^ | 1.30*10^-3^ | 5.57*10^-3^ | 1.47*10^-4^ | 1.21*10^-3^ | 7.17*10^-4^ |
|  | Terrestrial | 2.56*10^-2^ | 7.28*10^-4^ | 6.84*10^-4^ | 8.14*10^-3^ | 7.63*10^-4^ | 6.62*10^-4^ | 4.44*10^-3^ | 3.91*10^-3^ | 1.20*10^-3^ | 1.97*10^-3^ | 1.22*10^-3^ | 2.77*10^-3^ |
| Hydrological Region | | 2.10*10^-3^ | 1.83*10^-3^ | 1.50*10^-3^ | 7.94*10^-4^ | 1.87*10^-3^ | 2.04*10^-3^ | 3.57*10^-4^ | 7.04*10^-4^ | 8.39*10^-4^ | 1.06*10^-5^ | 8.50*10^-4^ | 1.12*10^-3^ |
| State | | 8.31*10^-3^ | 2.50*10^-3^ | 3.00*10^-4^ | 9.01*10^-3^ | 2.50*10^-3^ | 1.79*10^-3^ | 3.05*10^-3^ | 3.06*10^-3^ | 8.85*10^-3^ | 1.45*10^-3^ | 5.83*10^-3^ | 6.87*10^-3^ |
| USDA Region | | 5.77*10^-4^ | 1.92*10^-4^ | 4.08*10^-4^ | 3.07*10^-5^ | 1.30*10^-4^ | 8.57*10^-6^ | 9.61*10^-4^ | 2.17*10^-4^ | 1.08*10^-4^ | 9.35*10^-6^ | 4.27*10^-5^ | 9.25*10^-6^ |
| Water Type | |  |  |  |  |  |  |  |  |  |  |  |  |
|  | Fine-scale | -^c^ | 3.80*10^-4^ | -^c^ | 3.66*10^-3^ | 3.84*10^-4^ | 4.76*10^-3^ | -^c^ | 1.82*10^-3^ | -^c^ | 6.33*10^-4^ | 2.93*10^-4^ | 1.48*10^-3^ |
|  | Mid-scale | -^c^ | 0.00 | -^c^ | 8.12*10^-4^ | -^c^ | 1.18*10^-3^ | -^c^ | 6.91*10^-4^ | -^c^ | 2.31*10^-4^ | -^c^ | 2.97*10^-4^ |
|  | Coarse-scale | -^c^ | -^c^ | -^c^ | 1.54*10^-4^ | -^c^ | 0.00 | -^c^ | -^c^ | -^c^ | 4.40*10^-5^ | -^c^ | 1.17*10^-4^ |
|  | General | -^c^ | -^c^ | -^c^ | 4.95*10^-3^ | -^c^ | 3.57*10^-4^ | -^c^ | -^c^ | -^c^ | 5.81*10^-5^ | -^c^ | 1.86*10^-4^ |

^a^ Using GPS coordinates and county, samples were separately classified into regions using 14 different regional schemes, including schemes based on biome, two nested ecoregion schemes, aquatic and terrestrial habitat type, interstate climate region, hydrologic region (based on USGS HUC2 unit codes), three regional schemes used by US federal agencies, and four schemes based on agricultural practices and/or output. For US-specific regional schemes, sites outside the US were assigned to the region of the closest US site.

^b^ Freshwater status as well as four levels of water type were considered in this study. See footnote *h* in Supplemental Table 9 for a description of the four, nested water type classification schemes used here.

^c^  The – indicates that the values for the given feature were constant for all samples with data for the given target. As such, that feature was not included in the feature set for forest development.

**Supplemental Table 9:** Summary of factors considered methodological and non-methodological during analysis of datapoints. Note, not all non-methodological or methodological factors were used in all analyses. For example, waterway ID and site ID were not included in the variance partitioning analysis.

| **Methodological Factors Included in Analysis** | **Non-Methodological Factors Included in Analysis** | | |
| --- | --- | --- | --- |
| Sample Type (grab sample or Moore swab) | Spatial: | | Latitude, Longitude |
| Sample Filter Method (e.g., membrane filter, modified Moore swab, no filter) ° |  | Region | State, Region^c^ |
| Enumeration Method ^*a^ |  | Waterway | Waterway ID, Site ID, Water Type^d^, Freshwater Status^e^ |
| Volume ° | Temporal | | Month, Season, Year |
| Culture-based or Molecular Detection Method | Other | | Biome, Aquatic Habitat Type, Terrestrial Habitat Type |
| Target Gene (if molecular) |  | |  |
| Confirmation Method ° |  | |  |

^*^ Indicates available for fecal indicator bacteria count data only.

° Indicates available for pathogen presence-absence data only.

^a^ Four nested schemes were used for classifying fecal indicator bacteria enumeration method: fine, medium, coarse, and general. See text for more detail.

^b^ Canadian Province, Mexican State, US State/Territory, County (if not Canada, Mexico or US)

^c^ X regional schemes were used, including schemes based on predominant agriculture type, US administrative borders, ecoregion and climate. See text for more details.

^d^ Three nested schemes were used for classifying water type: fine, medium, and coarse.

^e^ Freshwater or brackish/saltwater.
